# Acute COVID-19 severity and impaired cognitive function up to 32 months after diagnosis: an observational study

**DOI:** 10.1186/s12916-026-04856-2

**Published:** 2026-04-11

**Authors:** Ingibjörg Magnúsdóttir, Anders B. Nygaard, Asle Hoffart, Gillian Murphy, Kadri Kõiv, Mary M. Barker, Anikó Lovik, Anna Bára Unnarsdóttir, Anna K. Kähler, Arna Hauksdóttir, Edda Bjork Thordardottir, Elías Eyþórsson, Elísabet U. Gísladóttir, Emily E. Joyce, Emma M. Frans, Gunnar Tómasson, Harpa Lind Hjördísar Jónsdóttir, Harpa Rúnarsdóttir, Hrönn Harðardóttir, John Arne Dahl, Jóhanna Jakobsdóttir, Karl Trygve Kalleberg, Kristjana Hrönn Ásbjörnsdóttir, Merete Ellingjord-Dale, Mette S. Istre, Nils Inge Landrø, Qing Shen, Ragnhild Bø, Reedik Mägi, Runólfur Pálsson, Sonja Hjellegjerde Brunvoll, Sverre Urnes Johnson, Arne Søraas, Fang Fang, Kelli Lehto, Omid V. Ebrahimi, Thor Aspelund, Unnur Anna Valdimarsdóttir

**Affiliations:** 1 Centre of Public Health Sciences, Faculty of Medicine, School of Health Sciences, University of Iceland, Reykjavik, Iceland; 2 Department of Microbiology, Oslo University Hospital, Oslo, Norway; 3 Modum Bad Psychiatric Center and Research Institute, Vikersund, Norway; 4 Institute of Environmental Medicine, Karolinska Institutet, Stockholm, Sweden; 5 Estonian Genome Centre, Institute of Genomics, University of Tartu, Tartu, Estonia; 6 Institute of Psychology, Leiden University, Leiden, The Netherlands; 7 Department of Medical Epidemiology and Biostatistics, Karolinska Institutet, Stockholm, Sweden; 8 Landspitali University Hospital, Reykjavik, Iceland; 9 Faculty of Medicine, School of Health Sciences, University of Iceland, Reykjavik, Iceland; 10 Faculty of Psychology, School of Health Sciences, University of Iceland, Reykjavik, Iceland; 11 Age Labs AS, Oslo, Norway; 12 Department of Psychology, University of Oslo, Oslo, Norway; 13 Clinical Research Center for Mental Disorders, Shanghai Pudong New Area Mental Health Center, Tongji University School of Medicine, Shanghai, China; 14 Institute for Advanced Study, Tongji University, Shanghai, China; 15 School of Public Health and General Practice Medicine, Tongji University, Shanghai, China; 16 Department of Experimental Psychology, University of Oxford, Oxford, UK; 17 The Icelandic Heart Association, Kopavogur, Iceland; 18 Department of Epidemiology, Harvard TH Chan School of Public Health, Boston, MA USA

**Keywords:** Cognitive function, Long covid, Brain fog, Cohort, COVID-19, Post-COVID-19 condition, Population

## Abstract

**Background:**

Cognitive dysfunction (“brain fog”) is a commonly reported post-COVID-19 symptom. Leveraging data from five general population cohorts across four European countries (Estonia, Iceland, Norway, and Sweden), we assessed long-term prevalence of impaired subjective cognitive function among individuals diagnosed with COVID-19 by acute illness severity.

**Methods:**

The included cohorts consisted of adult participants recruited from March 2020 and followed with self-report measures of cognitive function and past COVID-19 infection (except one cohort consisting of clinically confirmed COVID-19 cases) through February 2023. In a cross-sectional analysis we contrasted the prevalence of impaired cognitive function among individuals with and without a COVID-19 diagnosis, overall and by illness severity up to 32 months post-diagnosis. We adjusted for age, gender, education, relationship status, binge drinking, body mass index, previous psychiatric diagnosis, number of chronic medical conditions, and response period. In a longitudinal analysis, we assessed potential changes in cognitive function scores before and after COVID-19 diagnosis.

**Results:**

The study population consisted of 153,841 participants (71% women), with 31,359 (20.4%) reporting a positive COVID-19 test. Overall, a COVID-19 diagnosis was not statistically significantly associated with increased prevalence ratio (PR) of impaired cognitive function (PR 1.30 [95% CI: 0.98–1.71]). Individuals bedridden due to COVID-19 for 1–6 days (PR 1.38 [95% CI 0.96–1.99]) or ≥ 7 days (2.59 [1.55–4.33]) had higher prevalence of impaired cognitive function compared to those never diagnosed, while individuals never bedridden had a lower prevalence to those never diagnosed with COVID-19 (0.89 [0.80–1.00]). These findings were corroborated in the longitudinal analysis where a pre- to post diagnosis decline in cognitive function was observed among individuals bedridden due to COVID-19 (*p* < 0.0001).

**Conclusions:**

The data indicates that a severe COVID-19 acute illness course is associated with impaired cognitive function up to 18–32 months after COVID-19 diagnosis.

**Supplementary Information:**

The online version contains supplementary material available at 10.1186/s12916-026-04856-2.

## Background

Post-COVID-19 condition (PCC), also known as long COVID, has been defined as a cluster of symptoms that remain for 3 months or longer after the onset of COVID-19 [1]. Patients can experience a wide range of health problems, including pulmonary, cardiac, and neurological symptoms [2]. One of the most common symptoms of post-COVID-19 condition is “brain fog” or cognitive dysfunction, affecting about a fifth of those living with the condition [3, 4]. Cognitive dysfunction is a term that covers varying impairments in cognitive function, including the inability to concentrate, confusion, short-term memory loss, and dizziness [5, 6]. Historically, similar symptoms of cognitive dysfunction have been reported in earlier pandemics, including influenza pandemics (e.g. Russian influenza in the late nineteenth century and the Spanish flu pandemic in 1918–1919) [7].

Previous literature has reported a high prevalence of various symptoms of subjective and objective manifestations of cognitive impairment among individuals recovering from COVID-19 with these symptoms persisting throughout and after recovery [4, 6, 8–10]. While most existing studies have a maximum follow-up period of approximately 1 year, one study found that the increased risk of neurodegenerative disorders remained at 2 years among more than one million patients with recorded COVID-19 diagnoses of different severities from electronic health records, compared to patients with other respiratory conditions [11]. Although evidence shows that exposure to COVID-19 can have a substantial long-term effect on cognition, most studies have focused on hospitalised patients [4, 8] and/or have lacked appropriate comparison groups [4, 8]. Less is known about the extent to which subjective cognitive impairment remains a persistent feature among all COVID-19 patients in the general population, according to varying illness severity.

Leveraging data from five general population cohorts across four European countries, we aimed to study the prevalence of impaired subjective assessed cognitive function among individuals up to 32 months after COVID-19 diagnosis as compared with those not diagnosed. In line with our previous work [12, 13], we specifically explored the association between acute COVID-19 illness severity and impaired cognitive functioning across the 32-month follow-up period.

## Methods

### Study design and participants

This cross-sectional and longitudinal cohort study included five cohorts from four countries (Estonia, Iceland, Norway, and Sweden) with harmonised data collections prospectively planned in March 2020. These cohorts are all part of the COVIDMENT consortium, described elsewhere [14, 15]. The cohorts used different strategies for recruitment, including referral from already established cohorts, self-recruitment through social media, and other marketing strategies. Individuals aged 18 and older from cohorts were eligible to participate in the current study. The cohorts included in the present study are: The Estonian Biobank COVID-19 Cohort (EstBB-C19, *n* = 12,284), The Icelandic COVID-19 National Resilience Cohort (C19-Resilience, *n* = 23,966), The Norwegian COVID-19 Cohort (NCC, n = 136,818), The Norwegian COVID-19, Mental Health and Adherence Project (MAP-19, *n* = 11,059), and the Swedish Omtanke2020 study (*n* = 28,453). All participants provided written or electronic informed consent. All cohorts had ethical approvals from respective national or regional ethics committees (Additional File 1: Table S1) with varying numbers of data collection waves from March 27, 2020, to February 23, 2023. The dates for when data on cognitive function in each cohort were collected can be found in Additional File 1: Table S2.

### Measurements

Participants in all cohorts responded to web-based questionnaires. Identification of persons with a COVID-19 diagnosis was based on self-reports of a past confirmed positive reverse transcription polymerase chain reaction (RT-qPCR) test for all cohorts except for Omtanke2020, which also used positive antigen tests, and the NCC cohort, which relied on laboratory-confirmed SARS-CoV-2 (COVID-19) tests, as well as self-reports for positive tests reported after February 2022. To determine the severity of COVID-19, we used participants’ reports of the number of days they were bedridden due to COVID-19. Participants were asked “How many days were you confined to bed due to COVID-19 symptoms?” with the response options being “Never”, “One to six days”, and “Seven days or longer”. In EstBB-C19 only, self-reports of the number of days with fever, with the same response options, were used as a proxy for time bedridden. Time from first COVID-19 diagnosis until data collection was also determined by the duration between the reported date of COVID-19 diagnosis and the date of questionnaire completion. Time from diagnosis was categorised as 0 to 3 months (< 90 days), 3 to 6 months (90–179 days), 6 to 12 months (180–364 days), 12 to 18 months (365–544 days), and 18 to 32 months (≥ 545 days).

#### Compromised cognitive function

We used self-reports of impaired cognitive function, utilising different measures in each cohort with predefined cut-offs to determine impaired cognitive function. All available measurements of cognitive function for each individual were included in the analysis. In EstBB-C19, cognitive function was evaluated using a cognitive function questionnaire as part of the Mental Health Online Survey (Additional File 1: Table S3) [16]. The raw scores (Likert scale 1–5) were summed and impaired subjective cognitive function was defined as values at least 1.5 standard deviations (SD) below the mean of all EstBB-C19 participants. In the C19-Resilience cohort, the Patient-Reported Outcomes Measurement Information System (PROMIS®) short form (Adult version 2.0—Cognitive function 8a) was used to measure perceived cognitive function [17]. Raw scores were calculated for all participants and then translated into a *T*-score according to the PROMIS Cognitive Function scoring manual [18]. Individuals were regarded as having impaired cognitive function if their score was at least 1.5 SDs below the mean. In the NCC cohort, the Retrieval subscale of the Everyday Memory Questionnaire—Revised (EMQ-R) was used, with the recommended cut-off score of 2.68 which is defined as 2 SDs above the means for healthy controls [19]. The MAP-19 cohort assessed cognitive function using the Self-Report Webexec Questionnaire [20]. The scores can range from 6 to 24 and a score of 18 or above was used to define impaired cognitive function [20]. The Omtanke2020 cohort utilised two questionnaire items, asking participants “During the last four weeks, how bothered have you been by the following problems?”: “Difficulty concentrating” and “Difficulty thinking and/or remembering (brain fog)”, with response options ranging from 0 (“not at all”) to 2 (“very bothered”). Raw scores were calculated by summing the answers from the two items. Within-cluster resampling was carried out using the respective month of data collection to estimate monthly means and SDs. *Z*-scores for each response were generated using the participant's individual score and the respective monthly mean and SD. This was needed to account for multiple measurements for the participants within a 1- or 3-month period due to monthly data collection waves. Within-cluster resampling deals with correlated data (multiple measurements for a participant) by using a large number of samples taken with simple random sampling (with replacement for the different samples) [21]. This creates several datasets with exactly one measurement per participant. Each of these samples can be analysed separately and the results pooled to remove the clustering effect. We used this method to calculate means and standard deviations of cognitive function per month. To create *Z*-scores that are not biased by repeated measures, each *Z*-score was then calculated with the relevant monthly mean and SD. After obtaining the individual *Z*-scores, participants were defined as having impaired cognitive function if their *Z*-score was ≥ 1.5 SDs above the mean monthly *Z*-score.

### Statistical analysis

First, the distribution of sociodemographic and health-related factors of study participants was explored for each cohort individually and combined, along with symptom severity for individuals with a past COVID-19 diagnosis. We then performed a cross-sectional analysis comparing the prevalence of impaired cognitive function among individuals with and without a diagnosis of COVID-19, overall and in subgroup analyses by illness severity and time from diagnosis. In all analyses, first COVID-19 diagnosis was used. The subgroup analyses were not performed in the MAP-19 cohort due to the small number of COVID-19 cases, which limited the ability to generate estimates for subsets of the cohort in the subgroups.

Baseline data was used to determine covariates for all participants. We used robust (modified) Poisson regression models to estimate prevalence ratios (PRs) with 95% confidence intervals (CIs). In robust Poisson regression, a quasi-likelihood model can be applied to fit the data with a binary outcome [22]. The exponential of the effect size can be interpreted as a relative risk or a PR [23]. The estimates were adjusted for age and gender in the first model, and in the second model additionally for education, relationship status, binge drinking, body mass index (BMI), previous psychiatric diagnosis, number of chronic medical conditions, and response period (see Additional File 1: Table S4 for more details on covariates) [24]. Cohorts that lacked specific covariates excluded them from the respective models (Additional File 1: Table S4).

We further conducted stratified analyses among the cohorts with similar results patterns in the primary analysis (C19-Resilience, NCC, and Omtanke2020) and then separately in EstBB-C19. MAP-19 was again not included in the analysis due to the limited number of COVID-19 cases. We explored modification of the association between COVID-19 severity and cognitive function by gender, age (18–39 years, 40–59 years, or 60 years and older), education, symptoms of depression during the past 2 weeks (above threshold or below (information on depression not available in NCC)), history of psychiatric disorder diagnosis, and chronic medical condition. Additionally, we performed subgroup analysis among individuals diagnosed with COVID-19 (vs. never diagnosed) by year of infection (2020, 2021, or 2022), and number of infections (1 or ≥ 2).

To aggregate the cohort-specific results for all of the aforementioned analyses, random-effects meta-analyses were performed, using the metafor package in R, to estimate pooled PRs and 95% CIs [25]. Heterogeneity in the cohorts was examined using the *I*^2^ statistic [26].

Using data from the C19-Resilience cohort, we performed a comparison of repeated measures of PROMIS® Cognitive Function scores at two timepoints. Individuals were only included in this analysis if they had not been diagnosed with COVID-19 at the earlier timepoint. The participants were divided into four groups depending on if, by the second timepoint, they had been: not diagnosed with COVID-19, diagnosed with COVID-19 and not bedridden, diagnosed with COVID-19 and bedridden 1–6 days, or diagnosed with COVID-19 and bedridden ≥ 7 days. Focusing on changes between timepoints for individuals, a linear mixed-effects model approach was used to test the difference of cognitive function scores [27]. We adjusted for age, gender, education, number of chronic medical conditions, and previous psychiatric diagnosis. We used a repeated measures analysis of variance (RMANOVA) test to determine if there was a difference between the two timepoints and illness severity.

Statistical analyses were conducted in R (version 4.2.2). The study is reported according to the Strengthening the Reporting of Observational studies in Epidemiology checklist (Additional File 1: STROBE Statement).

## Results

After exclusion of study participants without assessment of cognitive function (*n* = 26,419) and missing information on COVID-19 (*n* = 2741) and covariates (*n* = 29,579), 153,841 participants were included for analysis (see Additional File: Fig. S1 for flowchart of study participants and Table S5 for comparison of included and excluded participants), with a total of 202,504 self-reported cognitive function assessments. In total, 31,359 (20.4%) participants tested positive for SARS-CoV-2 at least once during the study period (Table 1) representing 4–49% of participants in each cohort (Additional File 1: Table S6). Across the cohorts, a higher proportion of individuals were female (71.4%), and the mean age of participants was 50.1 years (SD = 14.4 years). The cohort-specific mean age ranged from 38.8 (MAP-19) to 56.2 years (C19-Resilience). In the cohorts that measured the following variables, a high proportion of study participants had a master’s or higher degree (44.3%), were non-smokers (52.7%) or former smokers (38.0%), and did not report binge drinking (80.7% (Additional File 1: Table S6)). History of psychiatric diagnosis was highest in EstBB C-19 (51.0%) and this ranged from 20–32% in the other cohorts (Additional File 1: Table S6). 6.4% of the responses were in the year 2020, 64.9% in 2021, 28.7% in 2022, and less than 0.1% in 2023. Individuals bedridden for at least 1 day due to their COVID-19 illness represented over half of the COVID-19 population (16,432 individuals, 52.4%). Approximately 65% of the COVID-19 study population were first diagnosed in December 2021 or later.

**Table 1:** Baseline characteristics of study participants in the five cohorts combined

	***N* = 153,841**
	***n* (%)**
Cohorts^a^	
EstBB C-19 (Estonia)	8002 (5.2)
C19-Resilience (Iceland)	15,590 (10.1)
MAP-19 (Norway)	3746 (2.4)
NCC (Norway)	108,905 (70.8)
Omtanke2020 (Sweden)	17,598 (11.4)
Gender	
Male	43,915 (28.5)
Female	109,893 (71.4)
Missing	33 (0.0)
Age (years)	
*Mean (SD)*	*50.1 (14*.*4)*
18–29	12,093 (7.9)
30–39	27,924(18.2)
40–49	35,233 (22.9)
50–59	37,047 (24.1)
60–69	27,306 (17.7)
70–100	14,238 (9.3)
Education	
Compulsory or less	4813 (3.1)
Upper secondary, vocational, or other	30,829(20.0)
Bachelor’s or diploma university degree	40,208 (26.1)
Master’s or PhD	60,366 (39.2)
Missing or not measured^b^	17,625 (11.5)
Relationship status	
Single	9332 (6.1)
In a relationship	27,438 (17.8)
Missing or not measured^b^	117,071 (76.1)
Body mass index (kg/m^2^)	
< 25	69,859 (45.4)
25–30	53,908 (35.0)
> 30	29,161 (19.0)
Missing or not measured^b^	913 (0.6)
Smoking status	
Never	77,750 (50.5)
Former smoker	53,908 (35.0)
Current smoker	13,667 (8.9)
Missing or not measured^b^	6311 (4.1)
Binge drinking	
No	30,475 (19.8)
Yes	7309 (4.8)
Missing or not measured^b^	116,057 (75.4)
History of psychiatric disorder	
No	29,540 (19.2)
Yes	14,940 (9.7)
Missing or not measured^b^	109,361 (71.1)
Chronic medical conditions	
No condition	102,384 (66.6)
One condition	35,220 (22.9)
Two conditions	9104 (5.9)
Three or more conditions	2553 (1.7)
Missing or not measured^b^	4580 (3.0)
Number of cognitive function measurements	
1	131,854 (85.7)
2	7404 (4.8)
3	7277 (4.7)
4	2474 (1.6)
5	4275 (2.7)
6	557 (0.4)
Response year^c^	
2020	12,856 (6.4)
2021	130,909 (64.9)
2022	57,886 (28.7)
2023	7 (0.0)
Number of COVID-19 diagnoses	
0	122,482 (79.6)
1	29,529 (19.2)
2 or more	1830 (1.2)
Time spent bedridden (first COVID-19 diagnosis)^d^	
Never bedridden	12,967 (41.4)
Bedridden 1–6 days	13,986 (44.6)
Bedridden 7 days or longer	2446 (7.8)
Missing	1960 (6.3)
Timing of first COVID-19 diagnosis^d^	
October 2020 or earlier	3030 (9.7)
November 2020–March 2021	4225 (13.5)
April 2021–November 2021	3654 (11.7)
December 2021 or later	20,450 (65.2)

^a^Abbreviations for cohort names: *EstBB-C19* The Estonian Biobank COVID-19 Cohort, *C19-Resilience* The Icelandic COVID-19 National Resilience Cohort, *NCC* The Norwegian COVID-19 Cohort, *MAP-19* The Norwegian COVID-19 Mental Health and Adherence Project

^b^See overview of specific cohorts with no measure of specific covariates in Additional File 1: Table S4

^c^Timing of response to questionnaires. All responses included, not only baseline

^d^Exclusively participants diagnosed with COVID-19

**Fig. 1:**
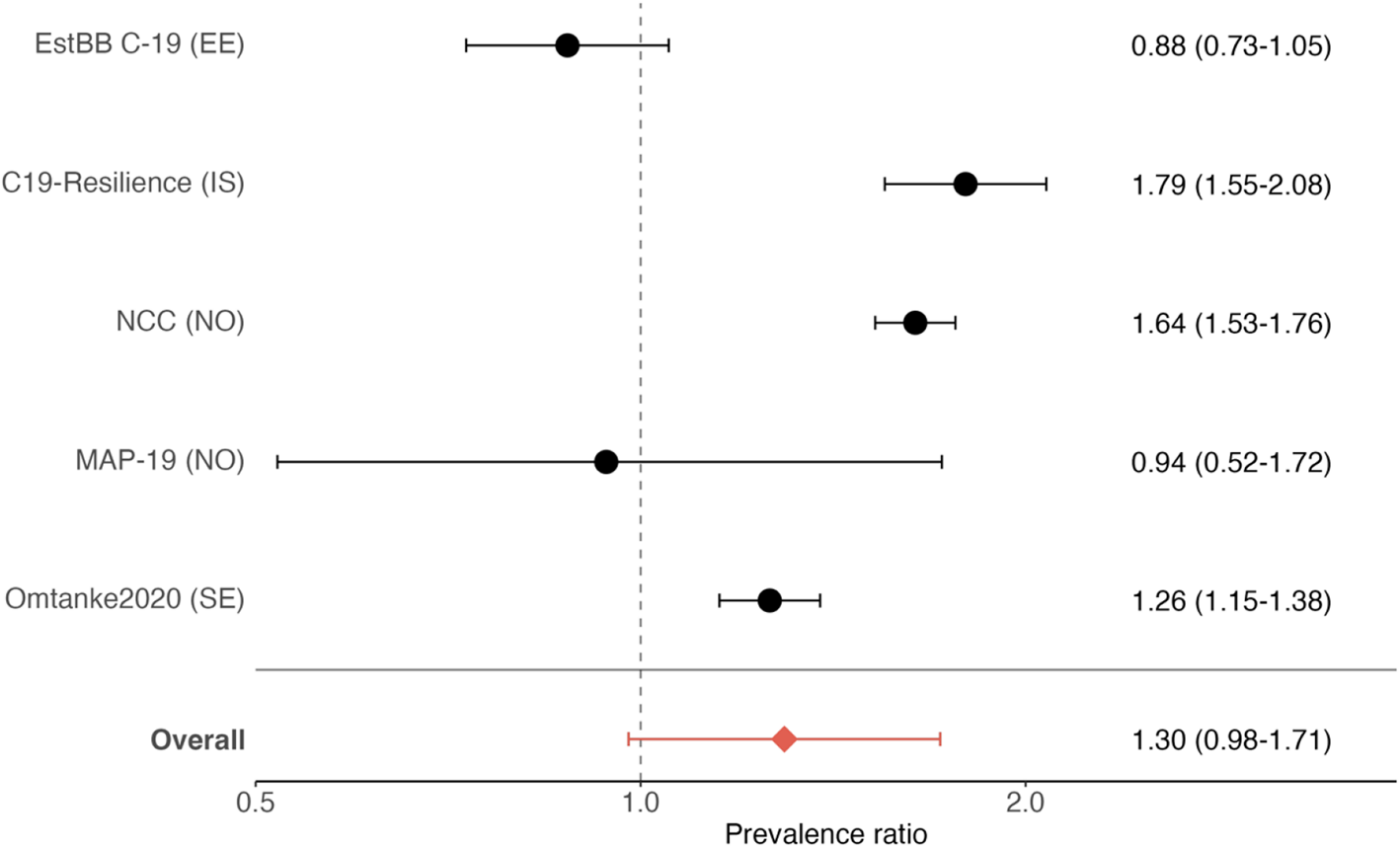
Prevalence ratios* (with 95% confidence intervals) of impaired cognitive function. *Multivariable adjusted

Figure 1 shows multivariable-adjusted PRs of impaired cognitive function among individuals diagnosed with COVID-19 compared with individuals without such a diagnosis, for each cohort and combined. While three out of five cohorts indicated an association between COVID-19 diagnosis and impaired cognitive function, the meta-analysis including all cohorts was not statistically significant (multivariable-adjusted PR 1.30, 95% CI 0.98–1.71; *I*^2^ 93.1%, *p* < 0.0001).

**Fig. 2:**
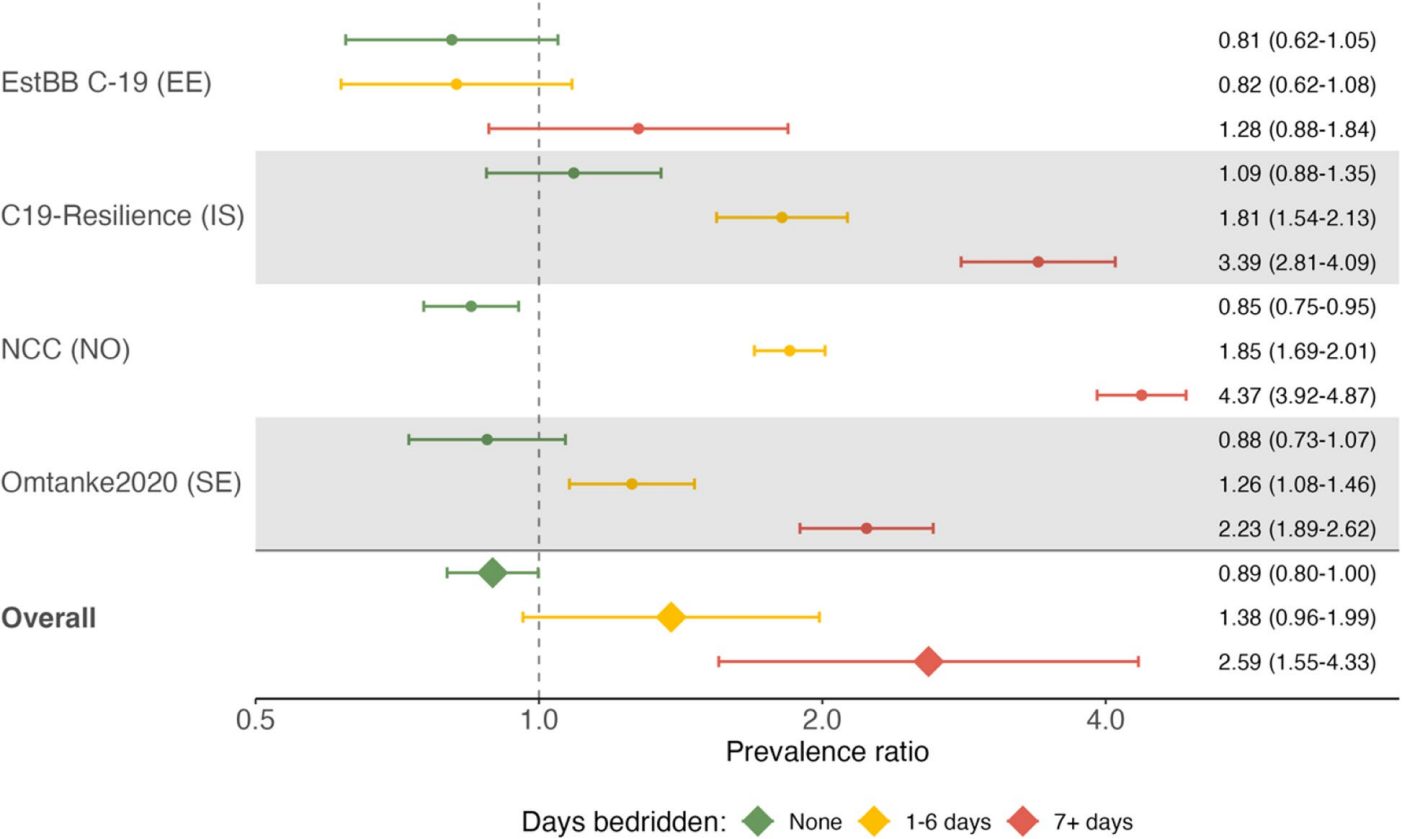
Prevalence ratios* (with 95% confidence intervals) of impaired cognitive function by illness severity (days bedridden). *Multivariable adjusted Note: MAP-19 is not included due to the small sample size

Longer time bedridden was associated with higher prevalence of impaired cognitive function in all cohorts, as well as the combined analysis. In the meta-analysis of all cohorts, when compared with those never diagnosed with COVID-19, individuals bedridden for 1–6 days had a higher prevalence of impaired function although not significant (PR 1.38 [95% CI 0.96–1.99]), while those bedridden ≥ 7 days (2.59 [1.55–4.33]) due to COVID-19 had a significantly higher prevalence of impaired cognitive function (Fig. 2 and Additional File 1: Table S7). Individuals never bedridden due to their illness had a significantly lower prevalence than those never diagnosed (PR 0.89 [95% CI 0.80–1.00]).

**Fig. 3:**
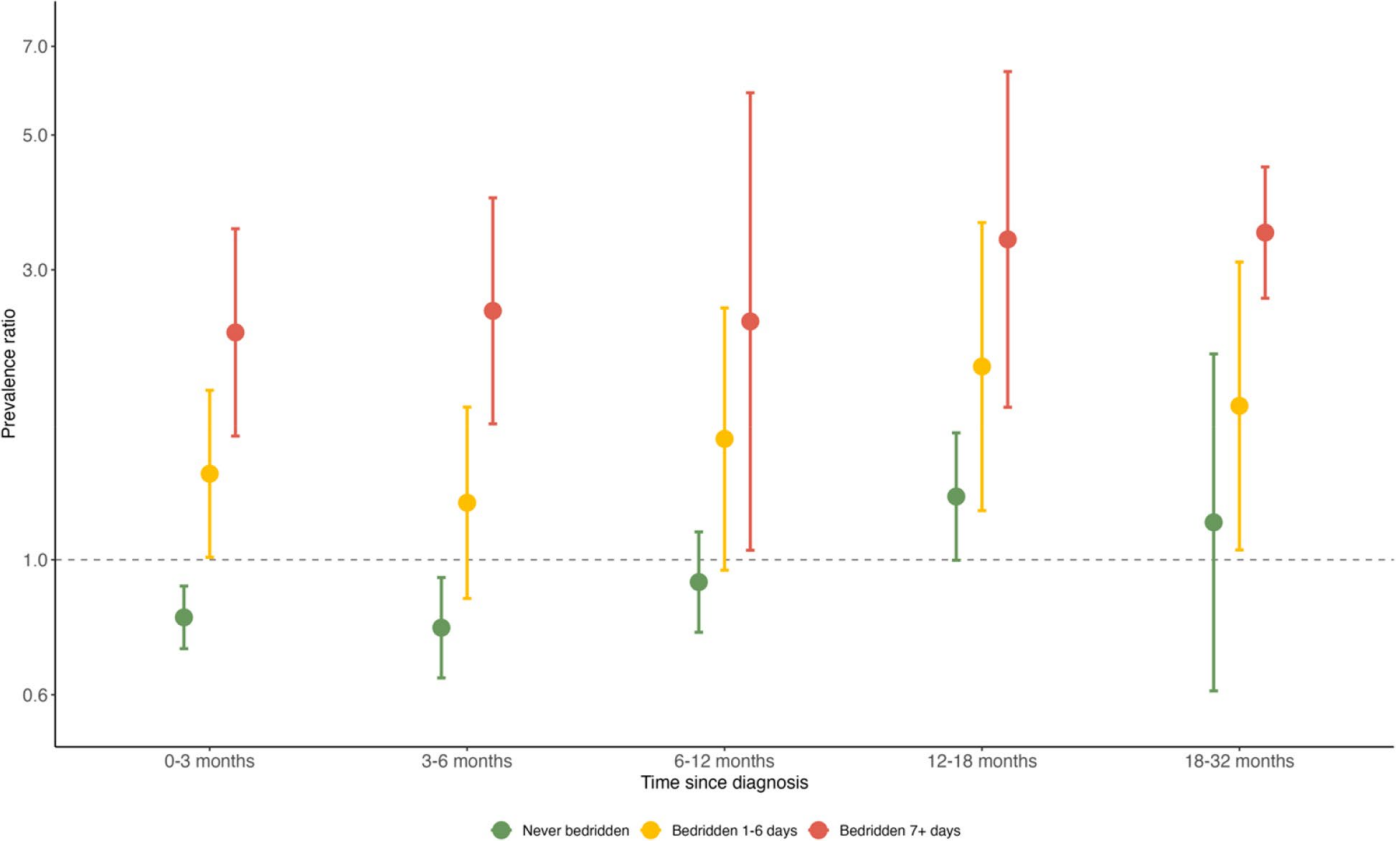
Prevalence ratios* (with 95% confidence intervals) of impaired cognitive function by time from diagnosis of COVID-19 by illness severity. *Multivariable adjusted Note: MAP-19 is not included due to the small sample size

The PRs of impaired cognitive function up to 32 months after first COVID-19 diagnosis according to time bedridden are shown in Fig. 3 and Additional File 1: Table S8. We found that the prevalence of impaired cognitive function did not decline with time from the diagnosis of COVID-19, particularly among individuals bedridden (Fig. 3 and Additional File 1: Table S8). Compared with participants without a COVID-19 diagnosis, individuals bedridden for ≥ 7 days showed a persistently higher prevalence of impaired cognitive function at 0–3 months (PR 2.37 [95% CI 1.60–3.50]), 3–6 months (2.57 [1.67–3.94]), 6–12 months (2.47 [1.04–5.87]), 12–18 months (3.37 [1.78–6.36]), and 18–32 months (3.45 [2.69–4.43]) after diagnosis. Individuals bedridden for 1–6 days due to COVID-19, compared with participants without a COVID-19 diagnosis, also persistently showed a higher prevalence of impaired cognitive function at 0–3 months (PR 1.38 [95% CI 1.01–1.90]), 3–6 months (1.24 [0.86–1.78]), 6–12 months (1.58 [0.96–2.60]), 12–18 months (2.08 [1.20–3.59]), and 18–32 months (1.79 [1.04–3.09]) following the diagnosis. By contrast, individuals never bedridden due to their COVID-19 illness did not show a significantly different prevalence of impaired cognitive function throughout most of the study period when compared with those without COVID-19, except for a marginally lower prevalence 0–6 months after diagnosis.

**Fig. 4:**
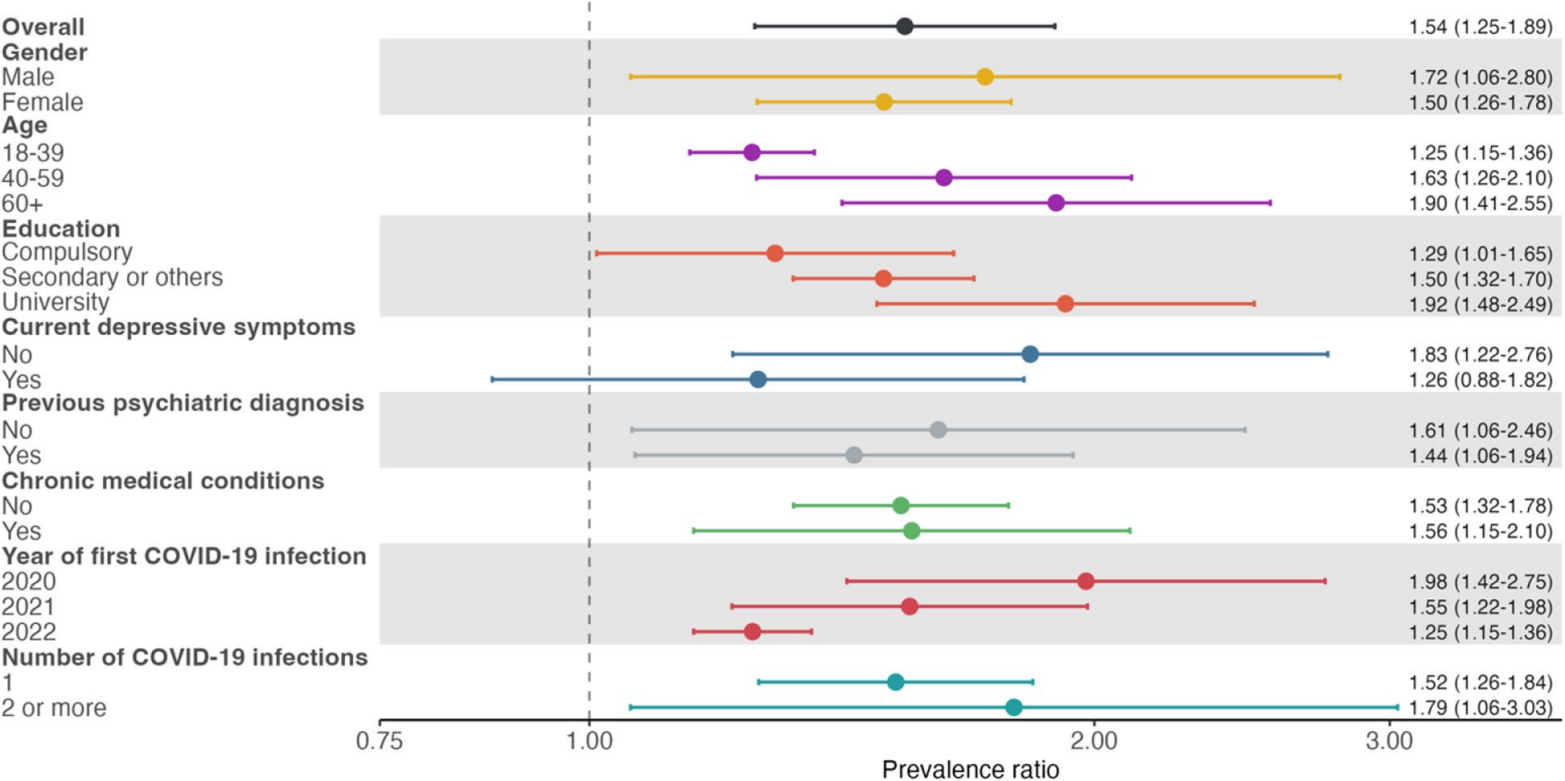
Impaired cognitive function stratified by participant characteristics Note: Stratification is limited to cohorts with similar result patterns in the overall analysis (C19-Resilience, NCC, and Omtanke2020). Symptoms of depression during the past 2 weeks measured using the Patient Health Questionnaire with the recommended cutoff score of ≥ 10 [29]

The association between COVID-19 diagnosis and impaired cognitive function in the analyses stratified by baseline characteristics is displayed in Fig. 4 and Additional File 1: Table S9 [28, 29]. Elevated prevalence of impaired cognitive function among those diagnosed with COVID-19 compared to those not diagnosed was observed in all subgroups. Age seemed to modify the association, as younger individuals (18–39 years) showed a weaker association between COVID-19 diagnosis and impaired cognitive function compared to the oldest group (1.25 [95% CI 1.15–1.36] vs. 1.90 [1.41–2.55], *p* = 0.02, respectively). Year of infection also modified the association, with a stronger association observed in 2020 compared to 2022 (1.98 [95% CI 1.42–2.75] vs. 1.25 [1.15–1.36], *p* = 0.008, respectively).

In the analysis of 6617 individuals from C19-Resilience with repeated assessments of cognitive function, the median time between measurements was 15.6 months (mean time 15.8 months) across all four groups (not diagnosed with COVID-19, diagnosed with COVID-19 and not bedridden, diagnosed with COVID-19 and bedridden 1–6 days, or diagnosed with COVID-19 and bedridden ≥ 7 days). Among individuals with a COVID-19 diagnosis, the time between diagnosis and the second measurement of cognitive function ranged from 7.3 to 7.7 months. Individuals never diagnosed with COVID-19 showed no difference in cognitive function between the two measurement points (difference = 0.2, *p* = 0.3; Table 2). By contrast, individuals diagnosed with COVID-19 but never bedridden, bedridden for 1–6 days, or bedridden for ≥ 7 days showed a decrease in adjusted mean score from the first measurement (before diagnosis) to the second measurement (after diagnosis): from 51.0 to 50.7 (difference = 0.3, *p* = 0.04), from 49.2 to 48.4 (difference = 0.6, *p* < 0.0001) and from 48.2 to 45.8 (difference = 2.4, *p* < 0.0001), respectively. The ANOVA test showed that the interaction between groups and timepoints was statistically significant (*p* < 0.0001).

**Table 2:** Mean PROMIS Cognitive Function scores among individuals with repeated measures

COVID-19 diagnosis	*n*	Mean number of months since diagnosis for last measurement	Cognitive function score^a^—first measurement (95% CI)	Cognitive function score^a^—last measurement (95% CI)	Change in cognitive function score (*p*-value of change)
No diagnosis	2021	–	50.1 (48.0–52.2)	49.9 (47.8–52.1)	− 0.2 (*p* = 0.3)
Bedridden 0 days	1808	7.6	51.0 (48.9–53.1)	50.7 (48.6–52.8)	− 0.3 (*p* = 0.04)
Bedridden 1–6 days	2562	7.3	49.2 (47.1–51.3)	48.4 (46.3–50.6)	− 0.6 (*p* < 0.0001)
Bedridden 7 + days	226	7.7	48.2 (45.9–50.6)	45.8 (43.4–48.2)	− 2.4 (*p* < 0.0001)

^a^Adjusted for age, gender, income, education, number of chronic medical conditions, and history of psychiatric disorders

## Discussion

In this study, that included over 150,000 individuals from the general population across four countries, we found that acute COVID-19 severity was associated with impaired subjective assessed cognitive function up to 32 months after diagnosis. While individuals diagnosed with COVID-19 who experienced mild acute illness (0 days bedridden) did not show increased prevalence of impaired cognitive function, we found that individuals bedridden for 7 days or longer due to COVID-19 persistently showed a two to three times elevated prevalence of impaired cognitive functioning compared to individuals not diagnosed with COVID-19 throughout the observation period. Similar results were observed in the longitudinal analysis.

The majority of existing literature indicates an association between COVID-19 and symptoms of impaired subjective and objective cognitive function [4, 6, 8, 9, 11]. To our knowledge, our study is the first study to address acute illness severity in relation to cognitive function in COVID-19 patients in the general population (i.e. not exclusively hospitalised patients) alongside a non-infected comparison group, with a follow-up period extending beyond 2 years. In contrast to previously reported findings [4, 8], our findings do not indicate that the SARS-CoV-2 infection itself is associated with impaired cognitive function. Indeed, infected individuals yet not bedridden showed similar (or lower) prevalence of impaired cognitive function while an increased number of days bedridden was associated with long-term elevated prevalence. These results are in line with our previous studies from the COVIDMENT consortium, in which time bedridden was shown to be associated with adverse mental and physical health outcomes in the long-term [12, 13]. Importantly, we found the symptoms of impaired cognitive function to be persistent, i.e. they did not attenuate with time among individuals bedridden during the acute phase of COVID-19. In line with our findings, studies on outbreaks of Severe Acute Respiratory Syndrome in 2003 and Middle East Respiratory Syndrome in 2015 have similarly reported a high prevalence of impaired concentration and memory 3 years after recovery from the infection [30]. Although it is possible that these adverse cognitive outcomes are not specific to severe COVID-19 illness and could be obtained after any severe illness, a recent study reported that COVID-19 presented with an increased risk of cognitive deficit 2 years after infection as compared to individuals with other infections [11].

The stratified analyses showed that the year of infection influenced the association between COVID-19 and impaired cognitive function, i.e. individuals infected in 2020 had a higher prevalence of impaired function, compared to those infected in 2022. This may reflect the existence of more potent SARS-CoV-2 variants circulating in 2020 than the Omicron variant, which was the most common variant in 2022 (and often not detected due to discontinuation of public screening measures) and the correspondingly more severe acute COVID-19 illness during the early pandemic [31, 32]. Also, COVID-19 vaccines were not yet available in 2020 [33] whereas between 77 and 90% of the populations in Iceland, Norway, and Sweden were vaccinated with at least one dose of a COVID-19 vaccine in 2022 [34].

Our longitudinal analysis, comparing cognitive function scores pre-to-post COVID-19 diagnosis, interestingly indicates that cognitive function scores may already be lower among those who ultimately suffered a more severe course of acute illness, possibly indicating an association between pre-infection levels of cognitive function and disease severity. Yet, this group of patients also distinctly experienced the largest decline in cognitive function scores from pre- to post- COVID-19 diagnosis. Regardless, larger, long-term follow-up studies are necessary to explore this association further.

Several hypotheses have been introduced to explain why some individuals suffer persistent symptoms after COVID-19, including impaired cognitive function. Different causes of post-COVID-19 condition have been proposed, including persistent viral components [35], abnormal blood clot formation due to hypercoagulability [36], autonomic nervous system dysfunction [37], and reduced serotonin levels [38]. However, sleep disruption or psychiatric disorders associated with COVID-19 could also negatively affect cognitive function [2]. Then again, we did not observe significant differences in point estimates by stratification nor by adjustment for previous history of psychiatric disorders.

Analyses within the MAP-19 and EstBB C-19 cohorts yielded different results from the other three cohorts. However, the MAP-19 cohort is very small, among which less than 4% of the participants reported being diagnosed with COVID-19 during the study period (Additional File 1: Table S6). Corroborating our previous findings [12], the EstBB C-19 cohort also showed differing results compared to the other cohorts in the present study. These discrepancies may be related to the selection of the EstBB C-19 study population, which consists solely of individuals tested for COVID-19 due to them being symptomatic before enrolment and were, therefore, tested. As a result, the control group in EstBB was affected by some other acute respiratory virus, potentially explaining the limited differences in cognitive outcomes between COVID-19 and non-COVID-19 participants, except for COVID-19 patients who suffered an extended acute illness course. We indeed noted an overall higher prevalence of impaired cognitive function in the control group of the EstBB C-19 cohort compared to the other cohorts (Additional File 1: Table S7) which may have been due to another infectious illness (as they were symptomatic).

The major strength of our study is the large sample size across four countries, including approximately 30 000 individuals who had been diagnosed with COVID-19 with varying disease severity, as well as a large comparison group. One of the main limitations relates to the different self-report tools to assess cognitive function in the different cohorts, which, as previously mentioned, may contribute to the variability in results obtained from the cohorts. However, the pattern of the results is similar in all cohorts with respect to the association between acute illness severity and prevalence of impaired cognitive function, namely that individuals with extended time bedridden consistently demonstrated the highest prevalence of cognitive impairment. The use of different measures to assess cognitive function in each cohort highlights one of the present challenges in this field of research, as these different measures may not uniformly capture the nuances of impaired cognition associated with SARS-CoV-2 infection. The use of different measures across different studies may hamper comparability and consistency of findings [39]. The use of self-report compared to objective testing has its limitations. Studies have shown that subjective memory rather than objective cognitive performance is more closely related to quality-of-life and mental health outcomes [40, 41]. Thus, self-reported cognitive dysfunction could reflect the mental state of the participants and also affect appraisal of acute COVID-19 severity. However, a history of psychiatric disorders was included in the multivariable models, and there was no clear effect modification of the associations by either the current levels of depressive symptoms or history of psychiatric disorders. Moreover, administering objective cognitive assessments on this scale would not have been feasible due to logistical and financial constraints. Another limitation relates to the cross-sectional study design; however, it was complemented with a longitudinal analysis of pre- to post-COVID-19 diagnosis change in cognitive function in the C19-Resilience cohort which yielded similar results. Finally, our study population comprised 70% female participants across the four countries which, along with other potential selection factors, may have affected the observed results. Yet, our stratified analyses indicate limited influence of gender on the estimates.

## Conclusions

In conclusion, this multinational study of individuals diagnosed with COVID-19 in four European general populations indicates that among individuals with COVID-19, acute illness severity is consistently associated with perceived impairment in cognitive function across all assessment periods, including among participants assessed up to 32 months after diagnosis. While more prospective data are needed, these findings motivate further research on the post-COVID-19 condition, with impaired cognitive function as a key feature, and highlight the need for monitoring of individuals experiencing severe acute COVID-19 illness.

## Supplementary Information

Additional file 1: Supplementary methods and Supplementary results, Tables S1-S9, Figure S1 and STROBE Statement. Table S1. Ethical approval for each cohort. Table S2. Dates of data collection for each cohort. Table S3. 4-item cognitive function questionnaire in the Mental Health Online survey. Table S4. Overview of available covariates included in statistical models by cohort. Figure S1. Flowchart for study with all cohorts and overall participation. Table S5. Baseline characteristics of study participants included and excluded from analysis. Table S6. Baseline characteristics of study participants in the five cohorts separately, combined, and combined with all missing and non-measured values removed. Table S7. The prevalence and prevalence ratios of impaired cognitive function among individuals with and without a diagnosis of COVID-19, including overall measures and subgroup analyses by illness severity and time from first diagnosis. Table S8. Prevalence ratio of impaired cognitive function during the first 2.5 years after diagnosis of COVID-19 by illness severity. Table S9. Prevalence and prevalence ratios of impaired cognitive function among individuals with and without a diagnosis of COVID-19, stratified by background characteristics.

## Data Availability

The individual-level data underlying this article cannot be shared publicly due to data protection laws in each participating country and GDPR.

## Abbreviations

BMI: Body mass index
C19-Resilience: COVID-19 National Resilience Cohort
CI: Confidence interval
EstBB-C19: Estonian Biobank COVID-19 Cohort
MAP-19: COVID-19, Mental Health and Adherence Project
NCC: Norwegian COVID-19 Cohort
PCC: Post-COVID-19 condition
PR: Prevalence ratio
RMANOVA: Repeated measures analysis of variance
RT-qPCR: Reverse transcription polymerase chain reaction
