# Supplementary material for: Acute COVID-19 severity and impaired cognitive function up to 32 months after diagnosis: an observational study
Source: BMC Med. 2026 Apr 11;24:311. doi: 10.1186/s12916-026-04856-2 (PMC13185386; doi:10.1186/s12916-026-04856-2)
Supplement: Supplementary file 1 — Additional file 1: Supplementary methods and Supplementary results, Tables S1-S9, Figure S1 and STROBE Statement. Table S1. Ethical approval for each cohort. Table S2. Dates of data collection for each cohort. Table S3. 4-item cognitive function questionnaire in the Mental Health Online survey. Table S4. Overview of available covariates included in statistical models by cohort. Figure S1. Flowchart for study with all cohorts and overall participation. Table S5. Baseline characteristics of study participants included and excluded from analysis. Table S6. Baseline characteristics of study participants in the five cohorts separately, combined, and combined with all missing and non-measured values removed. Table S7. The prevalence and prevalence ratios of impaired cognitive function among individuals with and without a diagnosis of COVID-19, including overall measures and subgroup analyses by illness severity and time from first diagnosis. Table S8. Prevalence ratio of impaired cognitive function during the first 2.5 years after diagnosis of COVID-19 by illness severity. Table S9. Prevalence and prevalence ratios of impaired cognitive function among individuals with and without a diagnosis of COVID-19, stratified by background characteristics. [file 12916_2026_4856_MOESM1_ESM.docx]

**Acute COVID-19 severity and impaired cognitive function up to 32 months after diagnosis: an observational study**

**Supplementary material**

Table of Contents

[Supplementary Methods 2](#_Toc226635391)

[Table S1: Ethical approval for each cohort 2](#_Toc226635392)

[Table S2: Dates of data collection for each cohort 3](#_Toc226635393)

[Table S3: 4-item cognitive function questionnaire in the Mental Health Online Survey 4](#_Toc226635394)

[Table S4: Overview of available covariates included in statistical models by cohort 5](#_Toc226635395)

[Supplementary Results 6](#_Toc226635396)

[Figure S1: Flowchart for study with all cohorts and overall participation 6](#_Toc226635397)

[Table S5: Baseline characteristics of study participants included and excluded from analysis 7](#_Toc226635398)

[Table S6: Baseline characteristics of study participants in the five cohorts separately, combined, and combined with all missing and non-measured values removed 11](#_Toc226635399)

[Table S7: The prevalence and prevalence ratios of impaired cognitive function among individuals with and without a diagnosis of COVID-19, including overall measures and subgroup analyses by illness severity and time from first diagnosis 15](#_Toc226635400)

[Table S8: Prevalence ratio of impaired cognitive function during the first 2.5 years after diagnosis of COVID-19 by illness severity. 20](#_Toc226635401)

[Table S9: Prevalence and prevalence ratios of impaired cognitive function among individuals with and without a diagnosis of COVID-19, stratified by background characteristics. 21](#_Toc226635402)

[STROBE Statement—Checklist of items that should be included in reports of *cross-sectional studies* 30](#_Toc226635403)

# **Supplementary Methods**

## Table S1: **Ethical approval for each cohort**

| **EstBB-C19**  Estonia | The activities of the EstBB are regulated by the Human Genes Research Act, which was adopted in 2000 specifically for the operations of the EstBB. Individual level data analysis in the EstBB was carried out under ethical approval [1.1-12/1277 and 1.1-12/212 25.01.2024.) from the Estonian Committee on Bioethics and Human Research (Estonian Ministry of Social Affairs), using data according to release application [P09-23/07/2021] from the Estonian Biobank. |
| --- | --- |
| **C19-Resilience**  Iceland | National Bioethics Committee (NBC 20-073, 21–071) as well as the National Data Protection Authority |
| **MAP-19**  Norway | Norwegian Regional Committee for Medical Research Ethics, reference number: 125510 |
| **NCC**  Norway | Norwegian Regional Committee for Research Ethics, reference number: REK 124170 |
| **Omtanke2020**  Sweden | Swedish Ethical Review Authority (DNR 2020-01785, DNR 2021-02835) |

## Table S2: **Dates of data collection for each cohort**

| **EstBB-C19**  Estonia | **C19-Resilience**  Iceland | **MAP-19**  Norway | **NCC**  Norway | **Omtanke2020**  Sweden |
| --- | --- | --- | --- | --- |
| April 2020-September 2021 | - April 2020-November 2020 - December 2020-March 2021 - May 2021-August 2021 - September 2022-January 2023 | November-December 2021 | December 2021-July 2022 | Repeated data collections from July 2021-February 2022 |

## Table S3: **4-item cognitive function questionnaire in the Mental Health Online Survey**

|  |  | ***Now we will look at how much you have been affected by the following issues in the LAST MONTH.*** | | | | | |
| --- | --- | --- | --- | --- | --- | --- | --- |
| **Nr** | **Item code** | **Question** | **Answer options** | | | | |
| *137* | *H5* | My mind is as sharp as usual | Completely disagree | Somewhat disagree | Neither disagree nor agree | Somewhat agree | Completely agree |
| *138* | *H6* | My memory is as good as usual | Completely disagree | Somewhat disagree | Neither disagree nor agree | Somewhat agree | Completely agree |
| *139* | *H7* | My thinking is as fast as usual | Completely disagree | Somewhat disagree | Neither disagree nor agree | Somewhat agree | Completely agree |
| *140* | *H8* | I can continue my activities consistently even if I am interrupted from time to time | Completely disagree | Somewhat disagree | Neither disagree nor agree | Somewhat agree | Completely agree |

## Table S4: **Overview of available covariates included in statistical models by cohort**

|  | **Classification of covariates** | **EstBB-C19**  Estonia | **C19-Resilience**  Iceland | **MAP-19**  Norway | **NCC**  Norway | **Omtanke2020**  Sweden |
| --- | --- | --- | --- | --- | --- | --- |
| Age | Discrete continuous, in years | × | × | × | × | × |
| Gender | Male or female | × | × | × | × | × |
| Education | Compulsory or less (no formal education); upper secondary, vocational, or other; bachelor's or diploma university degree; and master's or PhD | × | × | × | × | – |
| Relationship status | In a relationship or single | – | × | × | – | × |
| Binge drinking^a^ | Yes or no | × | × | – | – | × |
| Body mass index | <25, 25–30, or >30 kg/m2 | × | × | × | × | × |
| Previous psychiatric diagnosis**^b^** | Yes or no | × | × | × | – | × |
| Number of chronic medical conditions**^c^** | 0, 1, 2, or >2 conditions | × | × | – | × | × |
| Response period**^d^** | Three-month periods starting March 2020 and ending February 2023 | × | × | × | × | × |

Note: “×” denotes that covariate was included in models and “– “indicates that covariate was not included in models.

| ^a^ | Defined as having reported to have consumed alcohol usually 2—3 times or more per week in EstBB-C19 and consuming ≥5 drinks on at least one occasion for men or ≥4 drinks on at least one occasion for women in the last two months in C19-Resilience and last two weeks in Omtanke2020 |
| --- | --- |
| ^b^ | Obtained from electronic health records as any ICD-10 F-category diagnosis in EstBB-C19 and self-reported as yes or no in other cohorts |
| ^c^ | Chronic conditions included are hypertension, diabetes, heart disease, lung disease, chronic kidney disease, cancer, or immunosuppressive therapy in EstBB-C19, C19-Resilience, and NCC. In Omtanke2020 included chronic conditions are high blood pressure, heart disease, respiratory illness (including asthma), chronic kidney failure, cancer, diabetes, or impaired immune system due to other reasons. |
| ^d^ | March–May 2020, June–August 2020, September–November 2020, December 2020–February 2021, March–May 2021, June–August 2021, September–November 2021, December 2021–February 2022, March–May 2022, June–August 2022, September–November 2022, or December 2022–February 2023 |

# **Supplementary Results**


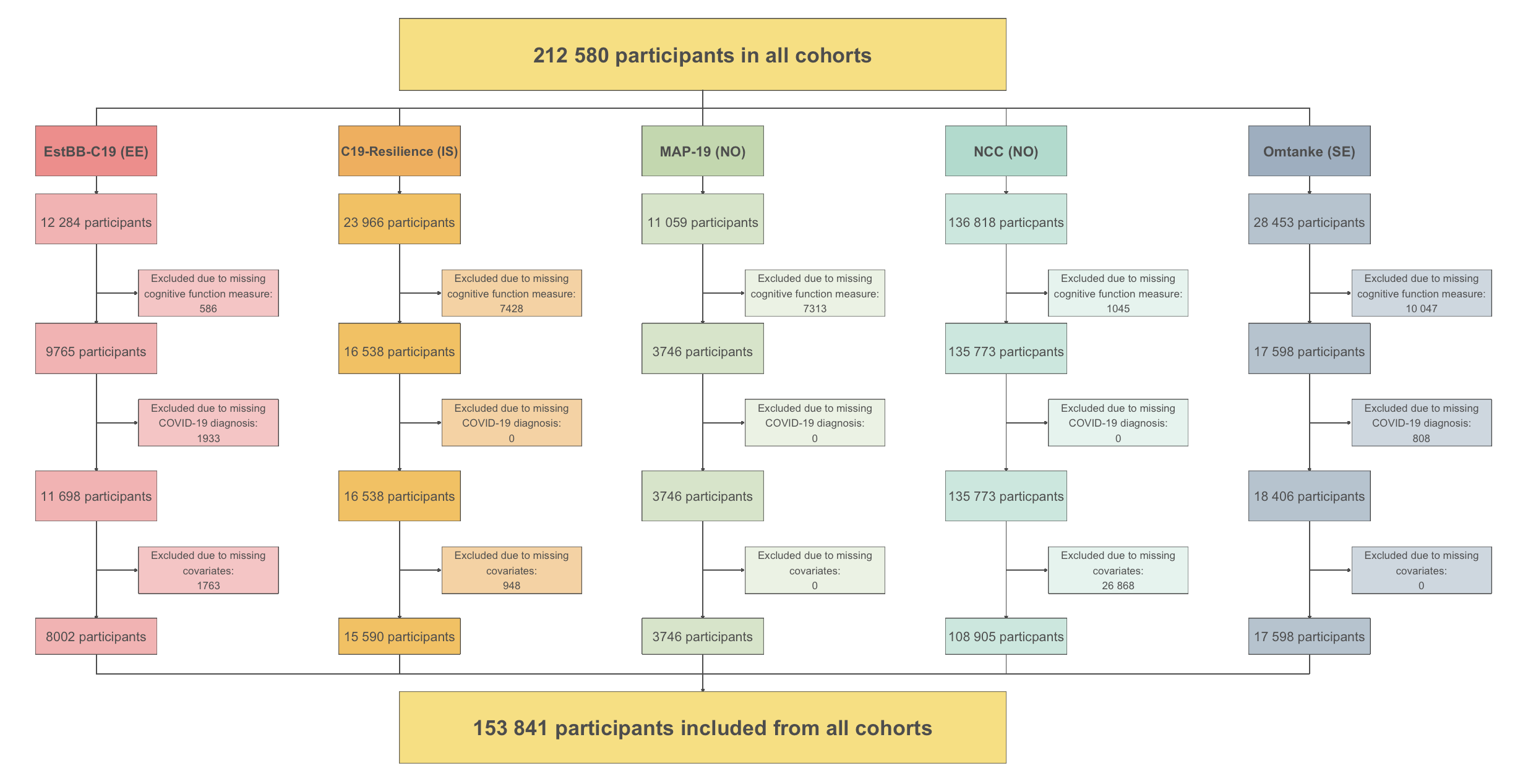


## Figure S1: **Flowchart for study with all cohorts and overall participation**

## Table S5: **Baseline characteristics of study participants included and excluded from analysis**

|  | **Estonia (EstBB C-19)** | | **Iceland**  **(C19-Resilience)** | | **Norway (NCC)** | | **Norway**  **(MAP-19)** | | **Sweden (Omtanke2020)** | |
| --- | --- | --- | --- | --- | --- | --- | --- | --- | --- | --- |
|  | **Included** | **Excluded** | **Included** | **Excluded** | **Included** | **Excluded** | **Included** | **Excluded** | **Included** | **Excluded** |
|  | **N = 8002** | **N = 4282** | **N = 15 590** | **N = 8376** | **N = 108 905** | **N = 27 913** | **N = 3746** | **N = 7313** | **N = 17 598** | **N = 10 855** |
|  | **n (%)** | **n (%)** | **n (%)** | **n (%)** | **n (%)** | **n (%)** | **n (%)** | **n (%)** | **n (%)** | **n (%)** |
| **Gender** | | | | | | | | | | |
| Male | 2344 (29.3) | 1329 (31.0) | 4744 (30.4) | 2302 (27.5) | 32 928 (30.2) | 9037 (32.4) | 748 (20.0) | 1436 (19.8) | 3151 (17.9) | 2115 (19.5) |
| Female | 5658 (70.7) | 2953 (69.0) | 10 846 (69.6) | 5519 (65.9) | 75 977 (69.8) | 18876 (67.6) | 2965 (79.2) | 5827 (80.2) | 14 447 (82.1) | 8740 (80.5) |
| Missing | - | - | - | 555 (6.6) | - | - | 33 (0.9) | - | - | - |
| ***Age (years)*** | | | | | | | | | | |
| *Mean (SD)* | *44.7 (13.2)* | *44.2 (13.7)* | *56.2 (13.9)* | *53.1 (15.4)* | *50 (14)* | *51 (14)* | *38.8 (14.0)* | *36 (13)* | *50.6 (15.4)* | *45.0 (16.0)* |
| 18-29 | 1008 (12.6) | 591 (13.8) | 700 (4.5) | 797 (9.5) | 7338 (6.7) | 2100 (7.5) | 1247 (33.3) | 2968 (40.8) | 1800 (10.2) | 2161 (20.0) |
| 30-39 | 2185 (27.3) | 1232 (28.8) | 1417 (9.1) | 907 (10.8) | 20 398 (18.7) | 4613 (16.5) | 942 (25.1) | 1819 (25.0) | 2982 (16.9) | 2270 (20.9) |
| 40-49 | 2058 (25.7) | 1077 (25.1) | 2540 (16.3) | 1493 (17.8) | 26 580 (24.4) | 6220 (22.3) | 690 (18.4) | 1202 (16.5) | 3365 (19.1) | 2080 (19.2) |
| 50-59 | 1612 (20.1) | 724 (16.9) | 3929 (25.2) | 2049 (24.5) | 27 059 (24.8) | 6980 (25.0) | 484 (12.9) | 811 (11.1) | 3963 (22.5) | 2110 (19.4) |
| 60-69 | 828 (10.3) | 456 (10.7) | 4238 (27.2) | 1939 (23.1) | 18 806 (17.3) | 5422 (19.4) | 290 (7.7) | 373 (5.1) | 3144 (17.9) | 1332 (12.3) |
| ≥70 | 311 (3.9) | 202 (4.7) | 2766 (17.7) | 1189 (14.2) | 8724 (8.0) | 2578 (9.2) | 93 (2.5) | 106 (1.5) | 2344 (13.3) | 902 (8.3) |
| Missing | - | - | *-* | 2 (<0.1) | - | - | - | - | - | - |
| **Education** | | | | | | | | | | |
| Compulsory or less | 129 (1.6) | 129 (3.0) | 2083 (15.4) | 1293 (15.4) | 2422 (2.2) |  | 179 (4.8) | 377 (5.2) | - | - |
| Upper secondary, vocational, or other | 2869 (35.9) | 1753 (40.9) | 4734 (30.4) | 2502 (29.9) | 21 925 (20.1) |  | 1301 (34.7) | 2872 (39.5) | - | - |
| Bachelor's or diploma university degree | 2217 (27.7) | 1204 (28.1) | 5056 (32.4) | 2258 (27.0) | 30 696 (28.2) |  | 2239 (59.8) | 4030 (55.4) | - | - |
| Master's or PhD | 2787 (34.8) | 1165 (27.2) | 3717 (23.8) | 1529 (18.3) | 53 862 (49.5) |  | - | - | - | - |
| Missing or not measured^a^ | - | 31 (0.7) | - | 794 (9.5) | - |  | 27 (0.7) | 34 (0.5) | 17 598 (100) | 10 855 (100) |
| **Relationship status** | | | | | | | | | | |
| Single | - | - | 3692 (23.7) | 1838 (21.9) | - | - | 1055 (28.2) | 2959 (40.5) | 4585 (26.1) | 3074 (28.3) |
| In a relationship | - | - | 11 898 (76.3) | 5945 (71.0) | - | - | 2691 (71.8) | 4320 (59.1) | 12 849 (73.0) | 7650 (70.5) |
| Missing or not measured^a^ | 8002 (100) | 4282 (100) | - | 593 (0.1) | 108 905 (100) | 27 913 (100) | - | 34 (0.5) | 164 (0.9) | 131 (1.2) |
| **Body-mass index (kg/m^2^)** | | | | | | | | | | |
| <25 | 3792 (47.4) | 1687 (39.4) | 4398 (28.2) | 2285 (27.3) | 50 672 (46.5) | 8994 (32.2) | 1732 (46.2) | - | 9265 (52.6) | 5199 (47.9) |
| 25-30 | 2558 (32.0) | 1232 (28.8) | 6057 (38.9) | 2881 (34.4) | 38 848 (35.7) | 3503 (12.5) | 1234 (32.9) | - | 5211 (29.6) | 2982 (27.5) |
| >30 | 1652 (20.6) | 777 (18.1) | 5135 (32.9) | 2112 (25.2) | 19 385 (17.8) | 7112 (25.5) | 780 (20.8) | - | 2209 (12.6) | 1566 (14.4) |
| Missing |  | 586 (13.7) | - | 1098 (13.1) | - | 8304 (29.7) | - | 7313 (100) | 913 (5.2) | 1108 (10.2) |
| **Current smoking** | | | | | | | | | | |
| Never | 4292 (53.6) | 2210 (51.6) | 7126 (45.7) | 3505 (41.8) | 57 026 (52.4) | 11 228 (41.1) | - | - | 9306 (52.9) | 5022 (46.2) |
| Former smoker | 2584 (32.3) | 1387 (32.4) | 5935 (38.1) | 2846 (34.0) | 42 078 (38.6) | 8757 (31.4) | - | - | 5516 (31.3) | 2977 (27.4) |
| Current smoker | 1117 (14.0) | 653 (15.2) | 2468 (15.8) | 1245 (14.9) | 7631 (7.0) | 1846 (6.6) | - | - | 2451 (13.9) | 2207 (20.3) |
| Missing or not measured^a^ | 9 (0.1) | 32 (0.8) | - | 780 (9.3) | 2170 (2.0) | 5828 (20.9) | 3746 (100) | 7313 (100) | 325 (1.8) | 649 (6.0) |
| **Binge drinking** | | | | | | | | | | |
| No | 6727 (84.1) | 3277 (76.5) | 14 025 (90.0) | 6717 (80.2) | - | - | - | - | 9723 (55.3) | 5262 (48.5) |
| Yes | 1275 (15.9) | 568 (13.3) | 1565 (10.0) | 880 (10.5) | - | - | - | - | 4469 (25.4) | 2815 (25.9) |
| Missing or not measured^a^ | - | 437 (10.2) | - | 779 (9.3) | 108 905 (100) | 27 913 (100) | 3746 (100) | 7313 (100) | 3406 (19.4) | 2778 (25.6) |
| **History of psychiatric disorder** | | | | | | | | | | |
| No | 3924 (49.0) | 1977 (46.2) | 11 082 (71.1) | 5122 (61.2) | - | - | 2998 (80.0) | - | 11 536 (65.6) | 6276 (57.8) |
| Yes | 4078 (51.0) | 2174 (50.8) | 4508 (28.9) | 2074 (24.8) | - | - | 748 (20.0) | - | 5606 (31.9) | 3868 (35.6) |
| Missing or not measured^a^ | - | 131 (3.1) | - | 1180 (14.1) | 108 905 (100) | 27 913 (100) | - | 7313 (100) | 456 (2.6) | 711 (6.6) |
| **Chronic medical conditions** | | | | | | | | | | |
| No condition | 4533 (56.6) | 2318 (54.1) | 8922 (57.2) | 4517 (53.9) | 77 470 (71.1) | 13 909 (49.8) | - | - | 11 459 (65.1) | 6720 (61.9) |
| One condition | 2531 (31.6) | 1080 (25.2) | 4632 (29.7) | 2144 (25.6) | 24 144 (22.2) | 5078 (18.2) | - | - | 3913 (22.2) | 2331 (21.5) |
| Two conditions | 673 (8.4) | 292 (6.8) | 1542 (9.9) | 741 (8.9) | 5832 (5.4) | 1369 (4.9) | - | - | 1057 (6.0) | 598 (5.5) |
| More than two conditions | 265 (3.3) | 135 (3.1) | 494 (3.2) | 163 (3.1) | 1459 (1.3) | 431 (1.5) | - | - | 335 (1.9) | 221 (2.0) |
| Missing or not measured^a^ | - | 457 (10.7) | - | 711 (8.5) | - | 7126 (25.5) | 3746 (100) | 7313 (100) | 834 (4.7) | 985 (9.1) |
| **Number of cognitive function measurements** | | | | | | | | | | |
| 0 | - | 586 (13.7) | - | 6663 (79.5) | - | 1045 (3.7) | - | 7313 (100) | 0 (0) | 10 047 (92.6) |
| 1 | 8002 (100) | 3696 (86.3) | 5040 (32.3) | 896 (10.7) | 108 905 (100) | 26 868 (96.3) | 3746 (100) | - | 6161 (35.0) | 460 (4.2) |
| 2 | - | - | 4846 (31.1) | 446 (5.3) | - | - | - | - | 2558 (14.5) | 28 (0.3) |
| 3 | - | - | 5704 (36.6) | 371 (4.4) | - | - | - | - | 1573 (8.9) | 27 (0.2) |
| 4 | - | - | - | - | - | - | - | - | 2474 (14.1) | 54 (0.5) |
| 5 | - | - | - | - | - | - | - | - | 4275 (24.3) | 196 (1.8) |
| 6 | - | - | - | - | - | - | - | - | 557 (3.2) | 43 (0.4) |
| **Number of COVID-19 diagnoses** | | | | | | | | | | |
| 0 | 6245 (78.0) | 2873 (67.1) | 8717 (55.9) | 6758 (80.7) | 89 693 (82.4) | 20 354 (72.9) | 3608 (96.3) | - | 14 218 (80.8) | 5185 (47.8) |
| 1 | 1757 (22.0) | 1251 (29.2) | 6528 (41.9) | 957 (11.4) | 18 687 (17.2) | 7363 (26.4) | 137 (3.7) | - | 3307 (18.8) | 810 (7.5) |
| 2 or more | - | - | 344 (2.2) | 49 (0.6) | 525 (0.5) | 196 (0.7) | 1 (0.0) | - | 73 (0.4) | 7 (<0.1) |
| Missing | - | 158 (3.7) | - | 612 (7.3) | - | - | - | 7313 (100) | - | 4853 (44.7) |

^a^ See an overview of which cohorts did not measure specific covariates in Table S4 p. 5

## Table S6: **Baseline characteristics of study participants in the five cohorts separately, combined, and combined with all missing and non-measured values removed**

|  | **Estonia (EstBB C-19)** | **Iceland**  **(C19-Resilience)** | **Norway (NCC)** | **Norway**  **(MAP-19)** | **Sweden (Omtanke2020)** | **Overall** | **Overall**  **(without missing and non-measured values)** |
| --- | --- | --- | --- | --- | --- | --- | --- |
|  | **N = 8002** | **N = 15 590** | **N = 108 905** | **N = 3746** | **N = 17 598** | **N = 153 841** | **N = 153 841** |
|  | **n (%)** | **n (%)** | **n (%)** | **n (%)** | **n (%)** | **n (%)** | **n (%)** |
| **Gender** | | | | | | | |
| Male | 2344 (29.3) | 4744 (30.4) | 32 928 (30.2) | 748 (20.0) | 3151 (17.9) | 43 915 (28.5) | 43 915 (28.6) |
| Female | 5658 (70.7) | 10 846 (69.6) | 75 977 (69.8) | 2965 (79.2) | 14 447 (82.1) | 109 893 (71.4) | 109 893 (71.4) |
| Missing | - | - | - | 33 (0.9) | - | 33 (0.0) | - |
| **Age (years)** | | | | | | | |
| *Mean (SD)* | *44.7 (13.2)* | *56.2 (13.9)* | *50 (14)* | *38.8 (14.0)* | *50.6 (15.4)* | *50.1 (14.4)* | *50.1 (14.4)* |
| 18-29 | 1008 (12.6) | 700 (4.5) | 7338 (6.7) | 1247 (33.3) | 1800 (10.2) | 12 093 (7.9) | 12 093 (7.9) |
| 30-39 | 2185 (27.3) | 1417 (9.1) | 20 398 (18.7) | 942 (25.1) | 2982 (16.9) | 27 924(18.2) | 27 924(18.2) |
| 40-49 | 2058 (25.7) | 2540 (16.3) | 26 580 (24.4) | 690 (18.4) | 3365 (19.1) | 35 233 (22.9) | 35 233 (22.9) |
| 50-59 | 1612 (20.1) | 3929 (25.2) | 27 059 (24.8) | 484 (12.9) | 3963 (22.5) | 37 047 (24.1) | 37 047 (24.1) |
| 60-69 | 828 (10.3) | 4238 (27.2) | 18 806 (17.3) | 290 (7.7) | 3144 (17.9) | 27 306 (17.7) | 27 306 (17.7) |
| ≥70 | 311 (3.9) | 2766 (17.7) | 8724 (8.0) | 93 (2.5) | 2344 (13.3) | 14 238 (9.3) | 14 238 (9.3) |
| **Education** | | | | | | | |
| Compulsory or less | 129 (1.6) | 2083 (15.4) | 2422 (2.2) | 179 (4.8) | - | 4813 (3.1) | 4813 (3.5) |
| Upper secondary, vocational, or other | 2869 (35.9) | 4734 (30.4) | 21 925 (20.1) | 1301 (34.7) | - | 30 829(20.0) | 30 829(22.6) |
| Bachelor's or diploma university degree | 2217 (27.7) | 5056 (32.4) | 30 696 (28.2) | 2239 (59.8) | - | 40 208 (26.1) | 40 208 (29.5) |
| Master's or PhD | 2787 (34.8) | 3717 (23.8) | 53 862 (49.5) | - | - | 60 366 (39.2) | 60 366 (44.3) |
| Missing or not measured^a^ | - | - | - | 27 (0.7) | 17 598 (100) | 17 625 (11.5) | - |
| **Relationship status** | | | | | | | |
| Single | - | 3692 (23.7) | - | 1055 (28.2) | 4585 (26.1) | 9332 (6.1) | 9332 (25.4) |
| In a relationship | - | 11 898 (76.3) | - | 2691 (71.8) | 12 849 (73.0) | 27 438(17.8) | 27 438(74.6) |
| Missing or not measured^a^ | 8002 (100) | - | 108 905 (100) | - | 164 (0.9) | 117 071 (76.1) | - |
| **Body-mass index (kg/m^2^)** | | | | | | | |
| <25 | 3792 (47.4) | 4398 (28.2) | 50 672 (46.5) | 1732 (46.2) | 9265 (52.6) | 69 859 (45.4) | 69 859 (45.7) |
| 25-30 | 2558 (32.0) | 6057 (38.9) | 38 848 (35.7) | 1234 (32.9) | 5211 (29.6) | 53 908(35.0) | 53 908(35.2) |
| >30 | 1652 (20.6) | 5135 (32.9) | 19 385 (17.8) | 780 (20.8) | 2209 (12.6) | 29 161 (19.0) | 29 161 (19.1) |
| Missing | - | - | - | - | 913 (5.2) | 913 (0.6) | - |
| **Current smoking** | | | | | | | |
| Never | 4292 (53.6) | 7126 (45.7) | 57 026 (52.4) | - | 9306 (52.9) | 77 750 (50.5) | 77 750 (52.7) |
| Former smoker | 2584 (32.3) | 5935 (38.1) | 42 078 (38.6) | - | 5516 (31.3) | 56 113 (36.5) | 56 113 (38.0) |
| Current smoker | 1117 (14.0) | 2468 (15.8) | 7631 (7.0) | - | 2451 (13.9) | 13 667 (8.9) | 13 667 (9.3) |
| Missing or not measured^a^ | 9 (0.1) | - | 2170 (2.0) | 3746 (100) | 325 (1.8) | 6311 (4.1) | - |
| **Binge drinking** | | | | | | | |
| No | 6727 (84.1) | 14 025 (90.0) | - | - | 9723 (55.3) | 30 475 (19.8) | 30 475 (80.7) |
| Yes | 1275 (15.9) | 1565 (10.0) | - | - | 4469 (25.4) | 7309 (4.8) | 7309 (19.3) |
| Missing or not measured^a^ | - | - | 108 905 (100) | 3746 (100) | 3406 (19.4) | 116 057 (75.4) | - |
| **History of psychiatric disorder** | | | | | | | |
| No | 3924 (49.0) | 11 082 (71.1) | - | 2998 (80.0) | 11 536 (65.6) | 29 540 (19.2) | 29 540 (19.2) |
| Yes | 4078 (51.0) | 4508 (28.9) | - | 748 (20.0) | 5606 (31.9) | 14 940(9.7) | 14 940(9.7) |
| Missing or not measured^a^ | - | - | 108 905 (100) | - | 456 (2.6) | 109 361 (71.1) | - |
| **Chronic medical conditions** | | | | | | | |
| No condition | 4533 (56.6) | 8922 (57.2) | 77 470 (71.1) | - | 11 459 (65.1) | 102 384 (66.6) | 102 384 (68.6) |
| One condition | 2531 (31.6) | 4632 (29.7) | 24 144 (22.2) | - | 3913 (22.2) | 35 220 (22.9) | 35 220 (23.6) |
| Two conditions | 673 (8.4) | 1542 (9.9) | 5832 (5.4) | - | 1057 (6.0) | 9104 (5.9) | 9104 (6.1) |
| More than two conditions | 265 (3.3) | 494 (3.2) | 1459 (1.3) | - | 335 (1.9) | 2553 (1.7) | 2553 (1.7) |
| Missing or not measured^a^ | - | - | - | 3746 (100) | 834 (4.7) | 4580 (3.0) | - |
| **Number of cognitive function measurements** | | | | | | | |
| 1 | 8002 (100) | 5040 (32.3) | 108 905 (100) | 3746 (100) | 6161 (35.0) | 131 854 (85.7) | 131 854 (85.7) |
| 2 | - | 4846 (31.1) | - | - | 2558 (14.5) | 7404 (4.8) | 7404 (4.8) |
| 3 | - | 5704 (36.6) | - | - | 1573 (8.9) | 7277 (4.7) | 7277 (4.7) |
| 4 | - | - | - | - | 2474 (14.1) | 2474 (1.6) | 2474 (1.6) |
| 5 | - | - | - | - | 4275 (24.3) | 4275 (2.7) | 4275 (2.7) |
| 6 | - | - | - | - | 557 (3.2) | 557 (0.4) | 557 (0.4) |
| **Response year^b^** | | | | | | | |
| 2020 | 2989 (37.4) | 9867 (32.5) | - | - | - | 12 856 (6.4) | 12 856 (6.4) |
| 2021 | 5013 (62.6) | 11 758 (38.7) | 65 886 (60.5) | 3746 (100) | 44 506 (87.9) | 130 909 (64.9) | 130 909 (64.9) |
| 2022 | - | 8764 (28.8) | 43 019 (39.5) | - | 6103 (12.1) | 57 886 (28.7) | 57 886 (28.7) |
| 2023 | - | 7 (0.0) | - | - | - | 7 (0.0) | 7 (0.0) |
| **Number of COVID-19 diagnoses** | | | | | | | |
| 0 | 6245 (78.0) | 8717 (55.9) | 89 693 (82.4) | 3608 (96.3) | 14 218 (80.8) | 122 482 (79.6) | 122 482 (79.6) |
| 1 | 1757 (22.0) | 6528 (41.9) | 18 687 (17.2) | 137 (3.7) | 3307 (18.8) | 29 529 (19.2) | 29 529 (19.2) |
| 2 or more | - | 344 (2.2) | 525 (0.5) | 1 (0.0) | 73 (0.4) | 1830 (1.2) | 1830 (1.2) |
| **Time spent bedridden (first COVID-19 diagnosis only) ^c^** | | | | | | | |
| Never bedridden | 866 (49.3) | 2533 (36.9) | 8949 (46.6) | 34 (24.6) | 585 (17.3) | 12 967 (41.4) | 12 967 (44.1) |
| Bedridden 1-6 days | 646 (36.8) | 3775 (54.9) | 8816 (45.9) | 70 (50.7) | 679 (20.1) | 13 986 (44.6) | 13 986 (47.6) |
| Bedridden 7 days or longer | 245 (13.9) | 564 (8.2) | 1447 (7.5) | 34 (24.6) | 156 (4.6) | 2446 (7.8) | 2446 (8.3) |
| Missing | - | - | - | - | 1960 (58.0) | 1960 (6.3) | - |
| **Timing of first COVID-19 diagnosis^c^** | | | | | | | |
| October 2020 or earlier | 418 (23.8) | 886 (12.9) | 1270 (6.7) | 12 (8.7) | 444 (13.1) | 3030 (9.7) | 3030 (9.7) |
| November 2020-March 2021 | 1 266 (72.1) | 314 (4.6) | 1288 (6.7) | 19 (13.8) | 1338 (39.6) | 4225 (13.5) | 4225 (13.5) |
| April 2021-November 2021 | 73 (4.2) | 431 (6.3) | 2526 (13.1) | 71 (51.4) | 553 (16.4) | 3654 (11.7) | 3654 (11.7) |
| December 2021 or later | - | 5241 (76.3) | 14 128 (73.5) | 36 (26.1) | 1045 (30.9) | 20 450 (65.2) | 20 450 (65.2) |

^a^ See an overview of which cohorts did not measure specific covariates in Table S4 p. 5

^b^ Timing of response to questionnaires. All responses included, not only baseline

^c^ Diagnosed with COVID-19 only

## Table S7: **The prevalence and prevalence ratios of impaired cognitive function among individuals with and without a diagnosis of COVID-19, including overall measures and subgroup analyses by illness severity and time from first diagnosis**

| **Estonia (Est-BBC19) N = 8002** | | | | **No confirmed diagnosis** | **Ever diagnosed with COVID-19** | **Never bedridden** | **Bedridden 1-6 days** | **Bedridden 7+ days** |  |
| --- | --- | --- | --- | --- | --- | --- | --- | --- | --- |
| **Overall** | n | | | 6 245 | 1757 | 866 | 646 | 245 |  |
|  | n_threshold_ (%) | | | 565 (9.0) | 134 (7.6) | 59 (6.8) | 48 (7.4) | 27 (11.0) |  |
|  | PR (crude)^a^ | | | Ref. | 0.84 (0.70-1.01) | 0.75 (0.58-0.98) | 0.81 (0.61-1.08) | 1.20 (0.83-1.74) |  |
|  | PR (age+gender adj.) ^a^ | | | Ref. | 0.86 (0.71-1.03) | 0.77 (0.59-1.00) | 0.81 (0.61-1.08) | 1.30 (0.90-1.87) |  |
|  | PR (multivar. adj.) ^a^ | | | Ref. | 0.88 (0.73-1.05) | 0.81 (0.62-1.05) | 0.82 (0.62-1.08) | 1.28 (0.88-1.84) |  |
| **Diagnosed with COVID-19 0-3 months ago** | n | | | - | 958 | 413 | 390 | 155 |  |
|  | n_threshold_ (%) | | | - | 77 (8.0) | 26 (6.3) | 32 (8.2) | 19 (12.3) |  |
|  | PR (crude)^a^ | | | - | 0.88 (0.69-1.11) | 0.69 (0.47-1.01) | 0.90 (0.64-1.27) | 1.34 (0.87-2.07) |  |
|  | PR (age+gender adj.) ^a^ | | | - | 0.88 (0.69-1.11) | 0.68 (0.46-1.00) | 0.88 (0.63-1.25) | 1.48 (0.96-2.28) |  |
|  | PR (multivar. adj.) ^a^ | | | - | 0.89 (0.71-1.13) | 0.71 (0.48-1.04) | 0.88 (0.62-1.24) | 1.50 (0.97-2.30) |  |
| **Diagnosed with COVID-19 3-6 months ago** | n | | | - | 427 | 223 | 167 | 37 |  |
|  | n_threshold_ (%) | | | - | 29 (6.8) | 13 (5.8) | 10 (6.0) | 6 (16.2) |  |
|  | PR (crude)^a^ | | | - | 0.74 (0.51-1.06) | 0.64 (0.38-1.10) | 0.66 (0.36-1.21) | 1.75 (0.83-3.66) |  |
|  | PR (age+gender adj.) ^a^ | | | - | 0.76 (0.53-1.09) | 0.67 (0.39-1.14) | 0.68 (0.37-1.24) | 1.71 (0.81-3.62) |  |
|  | PR (multivar. adj.) ^a^ | | | - | 0.78 (0.54-1.12) | 0.70 (0.41-1.20) | 0.71 (0.39-1.30) | 1.50 (0.69-3.23) |  |
| **Diagnosed with COVID-19 6-12 months ago** | n | | | - | 372 | 230 | 89 | 53 |  |
|  | n_threshold_ (%) | | | - | 28 (7.5) | 20 (8.7) | 6 (16.2) | 2 (3.8) |  |
|  | PR (crude)^a^ | | | - | 0.85 (0.59-1.24) | 0.99 (0.64-1.53) | 0.75 (0.35-1.64) | 0.42 (0.11-1.66) |  |
|  | PR (age+gender adj.) ^a^ | | | - | 0.91 (0.63-1.33) | 1.07 (0.69-1.66) | 0.77 (0.36-1.66) | 0.47 (0.12-1.86) |  |
|  | PR (multivar. adj.) ^a^ | | | - | 0.94 (0.65-1.37) | 1.14 (0.74-1.76) | 0.78 (0.36-1.70) | 0.45 (0.12-1.75) |  |
| **Diagnosed with COVID-19 12-18 months ago** | n | | | - | - | - | - | - |  |
|  | n_threshold_ (%) | | | - | - | - | - | - |  |
|  | PR (crude)^a^ | | | - | **-** | - | **-** | **-** |  |
|  | PR (age+gender adj.) ^a^ | | | - | **-** | - | **-** | **-** |  |
|  | PR (multivar. adj.) ^a^ | | | - | **-** | - | **-** | **-** |  |
| **Diagnosed with COVID-19 18-32 months ago** | n | | | - | - | - | - | - |  |
|  | n_threshold_ (%) | | | - | - | - | - | - |  |
|  | PR (crude)^a^ | | | - | **-** | **-** | **-** | **-** |  |
|  | PR (age+gender adj.) ^a^ | | | - | **-** | - | **-** | **-** |  |
|  | PR (multivar. adj.) ^a^ | | | - | **-** | **-** | **-** | **-** |  |
|  | | |  |  |  |  |  |  |  |
| **Iceland (C19-Resilience) N = 31 242** | | | | **No confirmed diagnosis** | **Ever diagnosed with COVID-19** | **Never bedridden** | **Bedridden 1-6 days** | **Bedridden 7+ days** |  |
| **Overall** | n | | | 22 738 | 7658 | 2844 | 4072 | 742 |  |
|  | n_threshold_ (%) | | | 1022 (4.5) | 582 (7.6) | 117 (4.1) | 342 (8.4) | 123 (16.6) |  |
|  | PR (crude)^a^ | | | Ref. | **2.03 (1.75-2.35)** | 1.05 (0.84-1.30) | **2.17 (1.83-2.56)** | **4.04 (3.37-4.85)** |  |
|  | PR (age+gender adj.) ^a^ | | | Ref. | **1.60 (1.38-1.85)** | 0.85 (0.69-1.05) | **1.68 (1.43-1.98)** | **3.48 (2.89-4.17)** |  |
|  | PR (multivar. adj.) ^a^ | | | Ref. | **1.79 (1.55-2.08)** | 1.09 (0.88-1.35) | **1.81 (1.54-2.13)** | **3.39 (2.81-4.09)** |  |
| **Diagnosed with COVID-19 0-3 months ago** | n | | | - | 204 | 78 | 87 | 39 |  |
|  | n_threshold_ (%) | | | - | 23 (11.3) | 5 (6.4) | 13 (14.9) | 5 (12.8) |  |
|  | PR (crude)^a^ | | | - | **2.44 (1.65-3.61)** | 1.42 (0.61-3.34) | **3.32 (2.00-5.51)** | **2.85 (1.25-6.48)** |  |
|  | PR (age+gender adj.) ^a^ | | | - | **1.92 (1.31-2.81)** | 1.14 (0.48-2.69) | **2.51 (1.57-4.00)** | **2.30 (1.03-5.12)** |  |
|  | PR (multivar. adj.) ^a^ | | | - | **1.85 (1.28-2.68)** | 1.31 (0.54-3.17) | **2.05 (1.32-3.18)** | **2.47 (1.13-5.38)** |  |
| **Diagnosed with COVID-19 3-6 months ago** | n | | | - | 1790 | 596 | 1034 | 160 |  |
|  | n_threshold_ (%) | | | - | 130 (7.3) | 24 (4.0) | 74 (7.2) | 32 (20.0) |  |
|  | PR (crude)^a^ | | | - | **1.91 (1.54-2.36)** | 1.00 (0.66-1.53) | **1.81 (1.38-2.38)** | **4.84 (3.48-6.73)** |  |
|  | PR (age+gender adj.) ^a^ | | | - | **1.56 (1.28-1.91)** | 0.83 (0.55-1.25) | **1.42 (1.10-1.83)** | **3.89 (2.85-5.32)** |  |
|  | PR (multivar. adj.) ^a^ | | | - | **1.68 (1.37-2.06)** | 1.02 (0.68-1.54) | **1.48 (1.15-1.91)** | **3.53 (2.51-4.96)** |  |
| **Diagnosed with COVID-19 6-12 months ago** | n | | | - | 4613 | 1747 | 2519 | 347 |  |
|  | n_threshold_ (%) | | | - | 317 (6.9) | 56 (3.2) | 198 (7.9) | 63 (18.2) |  |
|  | PR (crude)^a^ | | | - | **1.85 (1.55-2.20)** | 0.75 (0.56-1.01) | **1.84 (1.51-2.25)** | **4.18 (3.27-5.33)** |  |
|  | PR (age+gender adj.) ^a^ | | | - | **1.37 (1.15-1.64)** | **0.62 (0.46-0.83)** | **1.42 (1.16-1.75)** | **3.36 (2.62-4.32)** |  |
|  | PR (multivar. adj.) ^a^ | | | - | **1.62 (1.35-1.93)** | 0.84 (0.62-1.13) | **1.59 (1.30-1.95)** | **3.10 (2.41-4.00)** |  |
| **Diagnosed with COVID-19 12-18 months ago** | n | | | - | 444 | 179 | 183 | 82 |  |
|  | n_threshold_ (%) | | | - | 45 (10.1) | 13 (7.3) | 25 (13.7) | 7 (8.5) |  |
|  | PR (crude)^a^ | | | - | **2.49 (1.86-3.33)** | 1.67 (0.97-2.85) | **3.16 (2.15-4.66)** | 1.88 (0.92-3.85) |  |
|  | PR (age+gender adj.) ^a^ | | | - | **2.32 (1.74-3.10)** | 1.48 (0.88-2.49) | **2.96 (2.00-4.37)** | **2.09 (1.02-4.28)** |  |
|  | PR (multivar. adj.) ^a^ | | | - | **2.57 (1.90-3.46)** | 1.57 (0.91-2.72) | **3.30 (2.23-4.87)** | **2.46 (1.25-4.83)** |  |
| **Diagnosed with COVID-19 18-32 months ago** | n | | | - | 607 | 244 | 249 | 114 |  |
|  | n_threshold_ (%) | | | - | 67 (11.0) | 19 (7.8) | 32 (12.9) | 16 (14.0) |  |
|  | PR (crude)^a^ | | | - | **3.07 (2.33-4.05)** | **1.80 (1.12-2.88)** | **2.97 (2.05-4.31)** | **3.24 (1.99-5.30)** |  |
|  | PR (age+gender adj.) ^a^ | | | - | **2.47 (1.88-3.24)** | 1.42 (0.90-2.25) | **2.31 (1.61-3.32)** | **3.24 (2.00-5.27)** |  |
|  | PR (multivar. adj.) ^a^ | | | - | **2.98 (2.27-3.91)** | **1.92 (1.21-3.03)** | **2.50 (1.74-3.60)** | **3.88 (2.38-6.32)** |  |
|  | | |  |  |  |  |  |  |  |
| **Norway (NCC) N = 108 905** | | | | | **No confirmed diagnosis** | **Ever diagnosed with COVID-19** | **Never bedridden** | **Bedridden 1-6 days** | **Bedridden 7+ days** |
| **Overall** | | n | | | 89 693 | 19 212 | 8949 | 8816 | 1447 |
|  |  | n_threshold_ (%) | | | 3487 (3.9) | 1416 (7.4) | 329 (3.7) | 781 (8.9) | 306 (21.1) |
|  |  | PR (crude)^a^ | | | Ref. | **1.83 (1.70-1.96)** | 0.89 (0.79-1.00) | **2.13 (1.96-2.32)** | **5.26 (4.72-5.85)** |
|  |  | PR (age+gender adj.) ^a^ | | | Ref. | **1.59 (1.48-1.72)** | **0.80 (0.72-0.90)** | **1.81 (1.66-1.97)** | **4.68 (4.20-5.22)** |
|  |  | PR (multivar. adj.) ^a^ | | | Ref. | **1.64 (1.53-1.76)** | **0.85 (0.75-0.95)** | **1.85 (1.69-2.01)** | **4.37 (3.92-4.87)** |
| **Diagnosed with COVID-19 0-3 months ago** | | n | | | - | 11 675 | 5711 | 5441 | 523 |
|  |  | n_threshold_ (%) | | | - | 755 (6.5) | 206 (3.6) | 449 (8.3) | 100 (19.1) |
|  |  | PR (crude)^a^ | | | - | **1.55 (1.43-1.69)** | 0.86 (0.75-0.99) | **1.94 (1.75-2.15)** | **4.57 (3.80-5.50)** |
|  |  | PR (age+gender adj.) ^a^ | | | - | **1.34 (1.23-1.46)** | 0.76 (0.66-0.88) | **1.60 (1.44-1.78)** | **3.76 (3.12-4.54)** |
|  |  | PR (multivar. adj.) ^a^ | | | - | **1.38 (1.26-1.50)** | 0.80 (0.69-0.92) | **1.63 (1.47-1.81)** | **3.51 (2.90-4.24)** |
| **Diagnosed with COVID-19 3-6 months ago** | | n | | | - | 4449 | 2051 | 2142 | 256 |
|  |  | n_threshold_ (%) | | | - | 323 (7.3) | 74 (3.6) | 195 (9.1) | 54 (21.1) |
|  |  | PR (crude)^a^ | | | - | **1.60 (1.40-1.83)** | 0.75 (0.59-0.96) | **1.88 (1.58-2.23)** | **4.53 (3.52-5.83)** |
|  |  | PR (age+gender adj.) ^a^ | | | - | **1.33 (1.16-1.52)** | 0.64 (0.50-0.81) | **1.51 (1.27-1.79)** | **3.52 (2.73-4.55)** |
|  |  | PR (multivar. adj.) ^a^ | | | - | **1.44 (1.26-1.64)** | 0.72 (0.56-0.91) | **1.64 (1.38-1.95)** | **3.51 (2.72-4.53)** |
| **Diagnosed with COVID-19 6-12 months ago** | | n | | | - | 1156 | 464 | 470 | 222 |
|  |  | n_threshold_ (%) | | | - | 151 (13.1) | 22 (4.7) | 65 (13.8) | 64 (28.8) |
|  |  | PR (crude)^a^ | | | - | **3.31 (2.84-3.86)** | 1.18 (0.78-1.77) | **3.44 (2.74-4.32)** | **7.48 (6.06-9.23)** |
|  |  | PR (age+gender adj.) ^a^ | | | - | **2.96 (2.54-3.45)** | 1.08 (0.72-1.63) | **2.92 (2.32-3.67)** | **6.70 (5.42-8.28)** |
|  |  | PR (multivar. adj.) ^a^ | | | - | **2.95 (2.53-3.43)** | 1.13 (0.75-1.70) | **2.94 (2.34-3.70)** | **5.92 (4.78-7.32)** |
| **Diagnosed with COVID-19 12-18 months ago** | | n | | | - | 1009 | 424 | 409 | 176 |
|  |  | n_threshold_ (%) | | | - | 109 (10.8) | 18 (4.2) | 43 (10.5) | 48 (27.3) |
|  |  | PR (crude)^a^ | | | - | **2.75 (2.30-3.29)** | 1.08 (0.69-1.70) | **2.65 (1.99-3.53)** | **6.95 (5.45-8.85)** |
|  |  | PR (age+gender adj.) ^a^ | | | - | **2.46 (2.05-2.95)** | 0.98 (0.62-1.54) | **2.22 (1.66-2.96)** | **6.51 (5.09-8.31)** |
|  |  | PR (multivar. adj.) ^a^ | | | - | **2.48 (2.07-2.97)** | 1.03 (0.65-1.62) | **2.22 (1.66-2.97)** | **6.03 (4.73-7.68)** |
| **Diagnosed with COVID-19 18-32 months ago** | | n | | | - | 923 | 299 | 354 | 270 |
|  |  | n_threshold_ (%) | | | - | 78 (8.5) | 9 (3.0) | 29 (8.2) | 40 (14.8) |
|  |  | PR (crude)^a^ | | | - | **2.15 (1.74-2.67)** | 0.76 (0.40-1.45) | **2.07 (1.46-2.93)** | **3.79 (2.84-5.06)** |
|  |  | PR (age+gender adj.) ^a^ | | | - | **2.21 (1.79-2.74)** | 0.79 (0.41-1.49) | **1.98 (1.39-2.81)** | **4.19 (3.16-5.55)** |
|  |  | PR (multivar. adj.) ^a^ | | | - | **2.22 (1.79-2.74)** | 0.83 (0.44-1.56) | **2.04 (1.43-2.91)** | **3.88 (2.93-5.12)** |
|  | | |  |  |  |  |  |  |  |
|  | | |  |  |  |  |  |  |  |
| **Norway (MAP-19) N = 3746** | | | | | **No confirmed diagnosis** | **Ever diagnosed with COVID-19** | **Never bedridden** | **Bedridden 1-6 days** | **Bedridden 7+ days** |
| **Overall** | | n | | | 3 608 | 138 | 34 | 70 | 34 |
|  |  | n_threshold_ (%) | | | 389 (10.8) | 13 (9.6) | 3 (8.8) | 5 (7.1) | 5 (14.7) |
|  |  | PR (crude)^a^ | | | Ref. | 0.89 (0.50-1.60) | - | **-** | **-** |
|  |  | PR (age+gender adj.) ^a^ | | | Ref. | 0.88 (0.49-1.57) | **-** | **-** | **-** |
|  |  | PR (multivar. adj.) ^a^ | | | Ref. | 0.94 (0.52-1.72) | - | **-** | **-** |
| **Diagnosed with COVID-19 0-3 months ago** | | n | | | - | 36 | 11 | 17 | 8 |
|  |  | n_threshold_ (%) | | | - | 2 (5.6) | 0 (0.0) | 0 (0.0) | 2 (25.0) |
|  |  | PR (crude)^a^ | | | - | **-** | - | **-** | **-** |
|  |  | PR (age+gender adj.) ^a^ | | | - | **-** | **-** | **-** | **-** |
|  |  | PR (multivar. adj.) ^a^ | | | - | **-** | - | **-** | **-** |
| **Diagnosed with COVID-19 3-6 months ago** | | n | | | - | 16 | 4 | 7 | 5 |
|  |  | n_threshold_ (%) | | | - | 3 (18.8) | 1 (25.0) | 0 (0.0) | 2 (40.0) |
|  |  | PR (crude)^a^ | | | - | **-** | - | **-** | **-** |
|  |  | PR (age+gender adj.) ^a^ | | | - | **-** | - | **-** | **-** |
|  |  | PR (multivar. adj.) ^a^ | | | - | **-** | - | **-** | **-** |
| **Diagnosed with COVID-19 6-12 months ago** | | n | | | - | 55 | 14 | 32 | 9 |
|  |  | n_threshold_ (%) | | | - | 3 (5.5) | 1 (7.1) | 2 (6.3) | 0 (0.0) |
|  |  | PR (crude)^a^ | | | - | **-** | - | **-** | **-** |
|  |  | PR (age+gender adj.) ^a^ | | | - | **-** | - | **-** | **-** |
|  |  | PR (multivar. adj.) ^a^ | | | - | **-** | - | **-** | **-** |
| **Diagnosed with COVID-19 12-18 months ago** | | n | | | - | 19 | 3 | 10 | 6 |
|  |  | n_threshold_ (%) | | | - | 2 (10.5) | 1 (33.3) | 1 (10.0) | 0 (0.0) |
|  |  | PR (crude)^a^ | | | - | **-** | - | **-** | **-** |
|  |  | PR (age+gender adj.) ^a^ | | | - | **-** | - | **-** | **-** |
|  |  | PR (multivar. adj.) ^a^ | | | - | **-** | - | **-** | **-** |
| **Diagnosed with COVID-19 18-32 months ago** | | n | | | - | 12 | 2 | 4 | 6 |
|  |  | n_threshold_ (%) | | | - | 3 (25.0) | 0 (0.0) | 2 (50.0) | 1 (16.7) |
|  |  | PR (crude)^a^ | | | - | **-** | - | **-** | **-** |
|  |  | PR (age+gender adj.) ^a^ | | | - | **-** | - | **-** | **-** |
|  |  | PR (multivar. adj.) ^a^ | | | - | **-** | - | **-** | **-** |
|  | | |  |  |  |  |  |  |  |
|  | | |  |  |  |  |  |  |  |
| **Sweden (Omtanke2020) N = 50609** | | | | | **No confirmed diagnosis** | **Ever diagnosed with COVID-19** | **Never bedridden** | **Bedridden 1-6 days** | **Bedridden 7+ days** |
| **Overall** | | n | | | 42 984 | 7625^b^ | 1692^b^ | 1676^b^ | 938^b^ |
|  |  | n_threshold_ (%) | | | 3413 (7.9) | 882 (11.6) | 135 (8.0) | 207 (12.4) | 199 (21.2) |
|  |  | PR (crude)^a^ | | | Ref. | **1.38 (1.26-1.51)** | 0.96 (0.79-1.17) | **1.46 (1.24-1.71)** | **2.55 (2.16-3.01)** |
|  |  | PR (age+gender adj.) ^a^ | | | Ref. | **1.23 (1.13-1.35)** | 0.83 (0.69-1.00) | **1.25 (1.07-1.46)** | **2.44 (2.07-2.87)** |
|  |  | PR (multivar. adj.) ^a^ | | | Ref. | **1.26 (1.15-1.38)** | 0.88 (0.73-1.07) | **1.26 (1.08-1.46)** | **2.23 (1.89-2.62)** |
| **Diagnosed with COVID-19 0-3 months ago** | | n | | | - | 1216 | 502 | 554 | 100 |
|  |  | n_threshold_ (%) | | | - | 166 (13.7) | 47 (9.4) | 86 (15.5) | 28 (28.0) |
|  |  | PR (crude)^a^ | | | - | **1.40 (1.21-1.63)** | 0.98 (0.74-1.30) | **1.56 (1.26-1.94)** | **2.82 (2.07-3.85)** |
|  |  | PR (age+gender adj.) ^a^ | | | - | **1.21 (1.05-1.40)** | 0.85 (0.65-1.10) | **1.33 (1.09-1.63)** | **2.48 (1.81-3.40)** |
|  |  | PR (multivar. adj.) ^a^ | | | - | **1.18 (1.03-1.37)** | 0.85 (0.64-1.11) | **1.28 (1.05-1.57)** | **2.19 (1.59-3.03)** |
| **Diagnosed with COVID-19 3-6 months ago** | | n | | | - | 994 | 143 | 140 | 79 |
|  |  | n_threshold_ (%) | | | - | 92 (9.3) | 9 (6.3) | 10 (7.1) | 13 (16.5) |
|  |  | PR (crude)^a^ | | | - | 1.18 (0.97-1.43) | 0.85 (0.44-1.64) | 1.00 (0.53-1.88) | **2.04 (1.17-3.56)** |
|  |  | PR (age+gender adj.) ^a^ | | | - | 1.05 (0.87-1.28) | 0.72 (0.37-1.38) | 0.88 (0.47-1.67) | **1.93 (1.14-3.25)** |
|  |  | PR (multivar. adj.) ^a^ | | | - | 1.10 (0.90-1.34) | 0.77 (0.39-1.49) | 0.93 (0.49-1.78) | **1.71 (1.06-2.75)** |
| **Diagnosed with COVID-19 6-12 months ago** | | n | | | - | 3570 | 559 | 567 | 346 |
|  |  | n_threshold_ (%) | | | - | 394 (11.0) | 38 (6.8) | 67 (11.8) | 74 (21.4) |
|  |  | PR (crude)^a^ | | | - | **1.37 (1.21-1.55)** | 0.79 (0.54-1.16) | **1.39 (1.01-1.92)** | **2.68 (2.06-3.49)** |
|  |  | PR (age+gender adj.) ^a^ | | | - | **1.23 (1.09-1.39)** | **0.66 (0.45-0.98)** | 1.19 (0.86-1.63) | **2.56 (1.97-3.31)** |
|  |  | PR (multivar. adj.) ^a^ | | | - | **1.29 (1.15-1.46)** | 0.75 (0.51-1.11) | 1.29 (0.95-1.74) | **2.27 (1.76-2.93)** |
| **Diagnosed with COVID-19 12-18 months ago** | | n | | | - | 1304 | 388 | 306 | 279 |
|  |  | n_threshold_ (%) | | | - | 163 (12.5) | 37 (9.5) | 34 (11.1) | 57 (20.4) |
|  |  | PR (crude)^a^ | | | - | **1.50 (1.30-1.74)** | 1.28 (0.91-1.80) | **1.44 (1.02-2.03)** | **2.50 (1.91-3.28)** |
|  |  | PR (age+gender adj.) ^a^ | | | - | **1.36 (1.18-1.58)** | 1.11 (0.79-1.55) | 1.22 (0.87-1.72) | **2.42 (1.86-3.15)** |
|  |  | PR (multivar. adj.) ^a^ | | | - | **1.43 (1.23-1.66)** | 1.31 (0.94-1.83) | 1.24 (0.88-1.75) | **2.36 (1.80-3.09)** |
| **Diagnosed with COVID-19 18-32 months ago** | | n | | | - | 234 | 60 | 61 | 100 |
|  |  | n_threshold_ (%) | | | - | 36 (15.4) | 4 (6.7) | 5 (8.2) | 22 (22.0) |
|  |  | PR (crude)^a^ | | | - | **1.54 (1.14-2.09)** | 0.79 (0.31-2.03) | 0.94 (0.41-2.19) | **2.43 (1.62-3.64)** |
|  |  | PR (age+gender adj.) ^a^ | | | - | **1.42 (1.04-1.92)** | 0.68 (0.27-1.75) | 0.79 (0.35-1.79) | **2.54 (1.71-3.77)** |
|  |  | PR (multivar. adj.) ^a^ | | | - | **1.49 (1.09-2.03)** | 0.76 (0.28-2.06) | 0.83 (0.37-1.85) | **2.65 (1.79-3.92)** |

^a^ Significant prevalence ratios are in bold.

^b^ There is a discrepancy between the total number diagnosed with COVID-19 and the sum of individuals in the bedridden columns, due to missing data on the bedridden variable in the Omtanke2020 study.

## Table S8: **Prevalence ratio of impaired cognitive function during the first 2.5 years after diagnosis of COVID-19 by illness severity.**

| **Time since diagnosis** | **n** | **Time spent bedridden** | **PR (95% confidence interval)^a^** |
| --- | --- | --- | --- |
| 0-3 months | 6704 | Never bedridden | **0.80 (0.71-0.91)** |
|  | 6472 | Bedridden 1-6 days | **1.38 (1.01-1.90)** |
|  | 817 | Bedridden ≥7 days | **2.37 (1.60-3.50)** |
| 3-6 months | 3013 | Never bedridden | **0.77 (1.64-0.94)** |
|  | 3483 | Bedridden 1-6 days | 1.24 (0.86-1.78) |
|  | 532 | Bedridden ≥7 days | **2.57 (1.67-3.94)** |
| 6-12 months | 3000 | Never bedridden | 0.92 (0.76-1.11) |
|  | 3645 | Bedridden 1-6 days | 1.58 (0.96-2.60) |
|  | 968 | Bedridden ≥7 days | **2.47 (1.04-5.87)** |
| 12-18 months | 991 | Never bedridden | 1.27 (1.00-1.62) |
|  | 898 | Bedridden 1-6 days | **2.08 (1.20-3.59)** |
|  | 537 | Bedridden ≥7 days | **3.37 (1.78-6.36)** |
| 18-32 months | 603 | Never bedridden | 1.15 (0.61-2.18) |
|  | 664 | Bedridden 1-6 days | **1.79 (1.04-3.09)** |
|  | 484 | Bedridden ≥7 days | **3.45 (2.69-4.43)** |

^a^ Significant prevalence ratios are in bold.

## Table S9: **Prevalence and prevalence ratios of impaired cognitive function among individuals with and without a diagnosis of COVID-19, stratified by background characteristics.**

|  | **n** | **n_threshold_(%)** | **PR (crude)^a^** | **PR (age+gender adj.)^a^** | **PR (multivar. adj.)^a^** |
| --- | --- | --- | --- | --- | --- |
|  | **Estonia (EstBB-C19)** | | | | |
|  | N = 8002 | | | | |
| **No confirmed diagnosis** | 6 245 | 565 (9.0) | Ref. | Ref. | Ref. |
| **Ever diagnosed with COVID-19** | 1757 | 134 (7.6) | 0.84 (0.70-1.01) | 0.86 (0.71-1.03) | 0.88 (0.73-1.05) |
| ***Gender*** | | | | | |
| *Male* | | | | | |
| No confirmed diagnosis | 1743 | 118 (6.8) | Ref. | Ref. | Ref. |
| Ever diagnosed with COVID-19 | 601 | 29 (4.8) | 0.72 (0.48-1.08) | 0.74 (0.49-1.10) | 0.76 (0.51-1.14) |
| *Female* | | | | | |
| No confirmed diagnosis | 4 502 | 447 (9.9) | Ref. | Ref. | Ref. |
| Ever diagnosed with COVID-19 | 1156 | 105 (9.1) | 0.89 (0.72-1.09) | 0.89 (0.72-1.09) | 0.91 (0.74-1.12) |
| ***Age groups (years)*** | | | | | |
| *18-39* | | | | | |
| No confirmed diagnosis | 2481 | 292 (11.8) | Ref. | Ref. | Ref. |
| Ever diagnosed with COVID-19 | 712 | 66 (9.3) | 0.81 (0.63-1.05) | 0.81 (0.63-1.04) | 0.83 (0.65-1.07) |
| *40-59* | | | | | |
| No confirmed diagnosis | 2876 | 213 (7.4) | Ref. | Ref. | Ref. |
| Ever diagnosed with COVID-19 | 794 | 47 (5.9) | 0.77 (0.57-1.05) | 0.80 (0.59-1.09) | 0.81 (0.60-1.10) |
| *60+* | | | | | |
| No confirmed diagnosis | 888 | 60 (6.8) | Ref. | Ref. | Ref. |
| Ever diagnosed with COVID-19 | 251 | 21 (8.4) | 1.26 (0.78-2.04) | 1.25 (0.77-2.03) | 1.34 (0.84-2.12) |
| ***Education*** | | | | | |
| *Compulsory* | | | | | |
| No confirmed diagnosis | 96 | 11 (11.5) | Ref. | Ref. | Ref. |
| Ever diagnosed with COVID-19 | 33 | 7 (21.2) | 2.20 (0.77-6.29) | 2.10 (0.74-5.95) | 2.05 (0.80-5.25) |
| *Secondary or others* | | | | | |
| No confirmed diagnosis | 2164 | 192 (8.9) | Ref. | Ref. | Ref. |
| Ever diagnosed with COVID-19 | 705 | 48 (6.8) | 0.76 (0.56-1.03) | 0.78 (0.57-1.05) | 0.80 (0.59-1.09) |
| *University or above* | | | | | |
| No confirmed diagnosis | 3 985 | 362 (9.1) | Ref. | Ref. | Ref. |
| Ever diagnosed with COVID-19 | 1019 | 79 (7.8) | 0.85 (0.67-1.08) | 0.86 (0.68-1.10) | 0.89 (0.70-1.13) |
| ***Above threshold for depression (EST-Q2 instrument was used with the recommended cutoff score of >11)*** | | | | | |
| *No* | | | | | |
| No confirmed diagnosis | 4501 | 192 (4.3) | Ref. | Ref. | Ref. |
| Ever diagnosed with COVID-19 | 1329 | 56 (4.2) | 0.98 (0.73-1.32) | 1.00 (0.74-1.35) | 1.01 (0.75-1.36) |
| *Yes* |  |  |  |  |  |
| No confirmed diagnosis | 1717 | 369 (21.5) | Ref. | Ref. | Ref. |
| Ever diagnosed with COVID-19 | 421 | 78 (18.5) | 0.85 (0.68-1.06) | 0.85 (0.68-1.06) | 0.87 (0.70-1.08) |
| ***Previous psychiatric diagnosis*** | | | | | |
| *No* | | | | | |
| No confirmed diagnosis | 3 015 | 191 (6.3) | Ref. | Ref. | Ref. |
| Ever diagnosed with COVID-19 | 909 | 48 (5.3) | 0.82 (0.60-1.12) | 0.84 (0.61-1.15) | 0.84 (0.61-1.14) |
| *Yes* | | | | | |
| No confirmed diagnosis | 3230 | 374 (11.6) | Ref. | Ref. | Ref. |
| Ever diagnosed with COVID-19 | 848 | 86 (10.1) | 0.87 (0.70-1.09) | 0.89 (0.71-1.12) | 0.90 (0.72-1.13) |
| ***Pre-existing comorbidity*** | | | | | |
| *No* | | | | | |
| No confirmed diagnosis | 3 474 | 291 (8.4) | Ref. | Ref. | Ref. |
| Ever diagnosed with COVID-19 | 1059 | 76 (7.2) | 0.83 (0.65-1.06) | 0.85 (0.67-1.09) | 0.87 (0.68-1.11) |
| *Yes* | | | | | |
| No confirmed diagnosis | 2 771 | 274 (9.9) | Ref. | Ref. | Ref. |
| Ever diagnosed with COVID-19 | 698 | 58 (8.3) | 0.85 (0.65-1.12) | 0.88 (0.67-1.17) | 0.86 (0.65-1.14) |
| ***Year of COVID-19 diagnosis*** | | | | | |
| *2020* | | | | | |
| No confirmed diagnosis | - | - | Ref. | Ref. | Ref. |
| Ever diagnosed with COVID-19 | 769 | 200 (11.1) | 0.78 (0.60-1.02) | 0.81 (0.62-1.06) | 0.84 (0.64-1.10) |
| *2021* | | | | | |
| No confirmed diagnosis | - | - | Ref. | Ref. | Ref. |
| Ever diagnosed with COVID-19 | 988 | 47 (7.7) | 0.89 (0.70-1.12) | 0.89 (0.70-1.12) | 0.91 (0.72-1.15) |
| *2022* | | | | | |
| No confirmed diagnosis | - | - | - | - | - |
| Ever diagnosed with COVID-19 | - | - | **-** | - | - |
| ***Number of COVID-19 diagnoses*** | | | | | |
| *1* | | | | | |
| No confirmed diagnosis | - | - | Ref. | Ref. | Ref. |
| Ever diagnosed with COVID-19 | 1757 | 134 (7.6) | 0.84 (0.70-1.01) | 0.86 (0.71-1.03) | 0.88 (0.73-1.05) |
| *2 or more* | | | | | |
| No confirmed diagnosis | - | - | - | - | - |
| Ever diagnosed with COVID-19 | - | - | - | - | - |
|  | **Iceland (C19-Resilience)** | | | | |
|  | N = 30 654 | | | | |
| **No confirmed diagnosis** | 22 938 | 875 (3.8) | Ref. | Ref. | Ref. |
| **Ever diagnosed with COVID-19** | 8304 | 527 (6.3) | **2.03 (1.75-2.35)** | **1.60 (1.38-1.85)** | **1.79 (1.55-2.08)** |
| ***Gender*** | | | | | |
| *Male* | | | | | |
| No confirmed diagnosis | 7058 | 136 (1.9) | Ref. | Ref. | Ref. |
| Ever diagnosed with COVID-19 | 2409 | 104 (4.3) | **3.33 (2.43-4.57)** | **2.47 (1.79-3.41)** | **2.85 (2.06-3.93)** |
| *Female* | | | | | |
| No confirmed diagnosis | 15 680 | 886 (5.7) | Ref. | Ref. | Ref. |
| Ever diagnosed with COVID-19 | 5456 | 489 (9.0) | **1.84 (1.56-2.18)** | **1.45 (1.23-1.71)** | **1.61 (1.37-1.89)** |
| ***Age groups (years)*** | | | | | |
| *18-39* | | | | | |
| No confirmed diagnosis | 2731 | 302 (11.1) | Ref. | Ref. | Ref. |
| Ever diagnosed with COVID-19 | 1055 | 155 (14.7) | **1.36 (1.07-1.74)** | **1.45 (1.12-1.87)** | **1.42 (1.11-1.83)** |
| *40-59* | | | | | |
| No confirmed diagnosis | 9219 | 523 (5.7) | Ref. | Ref. | Ref. |
| Ever diagnosed with COVID-19 | 3385 | 340 (10.0) | **1.70 (1.38-2.10)** | **1.66 (1.35-2.06)** | **1.96 (1.60-2.42)** |
| *60+* | | | | | |
| No confirmed diagnosis | 10 788 | 197 (1.8) | Ref. | Ref. | Ref. |
| Ever diagnosed with COVID-19 | 3425 | 98 (2.9) | **1.73 (1.21-2.47)** | **1.61 (1.12-2.31)** | **2.02 (1.41-2.91)** |
| ***Education*** | | | | | |
| *Compulsory* | | | | | |
| No confirmed diagnosis | 3036 | 166 (5.5) | Ref. | Ref. | Ref. |
| Ever diagnosed with COVID-19 | 940 | 72 (7.7) | **1.42 (1.01-1.99)** | 1.04 (0.77-1.42) | 1.30 (0.94-1.81) |
| *Secondary or others* | | | | | |
| No confirmed diagnosis | 6974 | 321 (4.6) | Ref. | Ref. | Ref. |
| Ever diagnosed with COVID-19 | 2356 | 164 (7.0) | **1.76 (1.36-2.29)** | **1.31 (1.02-1.68)** | **1.48 (1.15-1.91)** |
| *University or above* | | | | | |
| No confirmed diagnosis | 12 728 | 535 (4.2) | Ref. | Ref. | Ref. |
| Ever diagnosed with COVID-19 | 4569 | 357 (7.8) | **2.39 (1.96-2.92)** | **2.06 (1.68-2.52)** | **2.23 (1.82-2.73)** |
| ***Above threshold for depression (using the Patient Health Questionnaire with the recommended cutoff score of ≥10)*** | | | | | |
| *No* | | | | | |
| No confirmed diagnosis | 19 215 | 208 (1.1) | Ref. | Ref. | Ref. |
| Ever diagnosed with COVID-19 | 6488 | 161 (2.5) | **2.71 (1.98-3.71)** | **2.19 (1.58-3.03)** | **2.33 (1.66-3.26)** |
| *Yes* |  |  |  |  |  |
| No confirmed diagnosis | 3523 | 814 (23.1) | Ref. | Ref. | Ref. |
| Ever diagnosed with COVID-19 | 1377 | 432 (31.4) | **1.53 (1.33-1.76)** | **1.44 (1.25-1.66)** | **1.52 (1.32-1.75)** |
| ***Previous psychiatric diagnosis*** | | | | | |
| *No* | | | | | |
| No confirmed diagnosis | 16 214 | 302 (1.9) | Ref. | Ref. | Ref. |
| Ever diagnosed with COVID-19 | 5682 | 223 (3.9) | **2.58 (1.98-3.37)** | **2.00 (1.53-2.62)** | **2.03 (1.55-2.66)** |
| *Yes* | | | | | |
| No confirmed diagnosis | 6524 | 720 (11.0) | Ref. | Ref. | Ref. |
| Ever diagnosed with COVID-19 | 2183 | 370 (16.9) | **1.82 (1.53-2.17)** | **1.59 (1.34-1.89)** | **1.69 (1.43-1.99)** |
| ***Pre-existing comorbidity*** | | | | | |
| *No* | | | | | |
| No confirmed diagnosis | 12 637 | 600 (4.7) | Ref. | Ref. | Ref. |
| Ever diagnosed with COVID-19 | 4625 | 374 (8.1) | **1.95 (1.63-2.34)** | **1.64 (1.37-1.98)** | **1.76 (1.46-2.12)** |
| *Yes* | | | | | |
| No confirmed diagnosis | 10 101 | 422 (4.2) | Ref. | Ref. | Ref. |
| Ever diagnosed with COVID-19 | 3240 | 219 (6.8) | **2.04 (1.60-2.60)** | **1.50 (1.19-1.91)** | **1.89 (1.50-2.39)** |
| ***Year of COVID-19 diagnosis*** | | | | | |
| *2020* | | | | | |
| No confirmed diagnosis | - | - | Ref. | Ref. | Ref. |
| Ever diagnosed with COVID-19 | 1805 | 200 (11.1) | **2.47 (2.13-2.85)** | **1.94 (1.67-2.25)** | **2.23 (1.93-2.59)** |
| *2021* | | | | | |
| No confirmed diagnosis | - | - | Ref. | Ref. | Ref. |
| Ever diagnosed with COVID-19 | 612 | 47 (7.7) | **1.76 (1.27-2.44)** | **1.58 (1.14-2.18)** | **1.64 (1.20-2.25)** |
| *2022* | | | | | |
| No confirmed diagnosis | - | - | Ref. | Ref. | Ref. |
| Ever diagnosed with COVID-19 | 5241 | 335 (6.4) | **1.48 (1.19-1.82)** | 1.14 (0.93-1.41) | **1.30 (1.06-1.60)** |
| ***Number of COVID-19 diagnoses*** | | | | | |
| *1* | | | | | |
| No confirmed diagnosis | - | - | Ref. | Ref. | Ref. |
| Ever diagnosed with COVID-19 | 7207 | 517 (7.2) | **1.95 (1.68-2.27)** | **1.56 (1.34-1.81)** | **1.75 (1.51-2.03)** |
| *2 or more* | | | | | |
| No confirmed diagnosis | - | - | Ref. | Ref. | Ref. |
| Ever diagnosed with COVID-19 | 530 | 69 (13.0) | **3.02 (2.26-4.02)** | **1.95 (1.46-2.60)** | **2.35 (1.76-3.14)** |
|  | **Norway (NCC)** | | | | |
|  | N = 109 209 | | | | |
| **No confirmed diagnosis** | 96 318 | 3874 (4.0) | Ref. | Ref. | Ref. |
| **Ever diagnosed with COVID-19** | 12 891 | 1039 (8.1) | **1.83 (1.70-1.96)** | **1.59 (1.48-1.72)** | **1.64 (1.53-1.76)** |
| ***Gender*** | | | | | |
| *Male* | | | | | |
| No confirmed diagnosis | 29 367 | 812 (2.8) | Ref. | Ref. | Ref. |
| Ever diagnosed with COVID-19 | 3666 | 180 (4.9) | **1.61 (1.37-1.90)** | **1.48 (1.25-1.75)** | **1.54 (1.30-1.81)** |
| *Female* | | | | | |
| No confirmed diagnosis | 66 951 | 3062 (4.6) | Ref. | Ref. | Ref. |
| Ever diagnosed with COVID-19 | 9225 | 859 (9.3) | **1.85 (1.71-2.00)** | **1.62 (1.49-1.76)** | **1.66 (1.53-1.80)** |
| ***Age groups (years)*** | | | | | |
| *18-39* | | | | | |
| No confirmed diagnosis | 23 720 | 1365 (5.8) | Ref. | Ref. | Ref. |
| Ever diagnosed with COVID-19 | 4102 | 335 (8.2) | **1.27 (1.13-1.44)** | **1.25 (1.11-1.41)** | **1.27 (1.13-1.43)** |
| *40-59* | | | | | |
| No confirmed diagnosis | 46 959 | 2089 (4.4) | Ref. | Ref. | Ref. |
| Ever diagnosed with COVID-19 | 6849 | 622 (9.1) | **1.79 (1.63-1.97)** | **1.73 (1.57-1.91)** | **1.77 (1.61-1.95)** |
| *60+* | | | | | |
| No confirmed diagnosis | 25 639 | 420 (1.6) | Ref. | Ref. | Ref. |
| Ever diagnosed with COVID-19 | 1940 | 82 (4.2) | **2.39 (1.86-3.08)** | **2.27 (1.77-2.91)** | **2.31 (1.80-2.96)** |
| ***Education*** | | | | | |
| *Compulsory* | | | | | |
| No confirmed diagnosis | 2214 | 188 (8.5) | Ref. | Ref. | Ref. |
| Ever diagnosed with COVID-19 | 215 | 26 (12.1) | **1.56 (1.09-2.23)** | 1.21 (0.83-1.76) | 1.27 (0.88-1.84) |
| *Secondary or others* | | | | | |
| No confirmed diagnosis | 19 667 | 1024 (5.2) | Ref. | Ref. | Ref. |
| Ever diagnosed with COVID-19 | 2322 | 233 (10.0) | **1.82 (1.59-2.10)** | **1.48 (1.29-1.71)** | **1.50 (1.30-1.73)** |
| *University or above* | | | | | |
| No confirmed diagnosis | 74 437 | 2662 (3.6) | Ref. | Ref. | Ref. |
| Ever diagnosed with COVID-19 | 10 354 | 780 (7.5) | **1.88 (1.73-2.05)** | **1.69 (1.55-1.84)** | **1.71 (1.57-1.86)** |
| ***Above threshold for depression*** | | | | | |
| *No* | | | | | |
| No confirmed diagnosis | - | - | - | - | - |
| Ever diagnosed with COVID-19 | - | - | - | - | - |
| *Yes* |  |  |  |  |  |
| No confirmed diagnosis | - | - | - | - | - |
| Ever diagnosed with COVID-19 | - | - | - | - | - |
| ***Previous psychiatric diagnosis*** | | | | | |
| *No* | | | | | |
| No confirmed diagnosis | - | - | - | - | - |
| Ever diagnosed with COVID-19 | - | - | - | - | - |
| *Yes* | | | | | |
| No confirmed diagnosis | - | - | - | - | - |
| Ever diagnosed with COVID-19 | - | - | - | - | - |
| ***Pre-existing comorbidity*** | | | | | |
| *No* | | | | | |
| No confirmed diagnosis | 67 885 | 2590 (3.8) | Ref. | Ref. | Ref. |
| Ever diagnosed with COVID-19 | 9802 | 727 (7.4) | **1.74 (1.59-1.90)** | **1.55 (1.41-1.69)** | **1.58 (1.45-1.73)** |
| *Yes* | | | | | |
| No confirmed diagnosis | 6679 | 337 (5.0) | Ref. | Ref. | Ref. |
| Ever diagnosed with COVID-19 | 629 | 72 (11.4) | **2.12 (1.87-2.40)** | **1.72 (1.51-1.95)** | **1.75 (1.54-1.99)** |
| ***Year of COVID-19 diagnosis*** | | | | | |
| *2020* | | | | | |
| No confirmed diagnosis | - | - | Ref. | Ref. | Ref. |
| Ever diagnosed with COVID-19 | 1826 | 181 (9.9) | **2.55 (2.21-2.93)** | **2.42 (2.10-2.79)** | **2.44 (2.12-2.81)** |
| *2021* | | | | | |
| No confirmed diagnosis | - | - | Ref. | Ref. | Ref. |
| Ever diagnosed with COVID-19 | 3371 | 322 (9.6) | **2.02 (1.84-2.22)** | **1.80 (1.63-1.98)** | **1.83 (1.66-2.01)** |
| *2022* | | | | | |
| No confirmed diagnosis | - | - | Ref. | Ref. | Ref. |
| Ever diagnosed with COVID-19 | 7694 | 536 (7.0) | **1.47 (1.33-1.63)** | **1.20 (1.09-1.33)** | **1.26 (1.14-1.39)** |
| ***Number of COVID-19 diagnoses*** | | | | | |
| *1* | | | | | |
| No confirmed diagnosis | - | - | Ref. | Ref. | Ref. |
| Ever diagnosed with COVID-19 | 12 436 | 987 (7.9) | **1.80 (1.67-1.94)** | **1.57 (1.46-1.69)** | **1.62 (1.50-1.74)** |
| *2 or more* | | | | | |
| No confirmed diagnosis | - | - | Ref. | Ref. | Ref. |
| Ever diagnosed with COVID-19 | 455 | 52 (11.4) | **1.80 (1.67-1.94)** | **1.57 (1.46-1.69)** | **1.62 (1.50-1.74)** |
|  | **Sweden (Omtanke2020)** | | | | |
|  | N = 50 609 | | | | |
| **No confirmed diagnosis** | 42 984 | 3413 (7.9) | Ref. | Ref. | Ref. |
| **Ever diagnosed with COVID-19** | 7625 | 882 (11.6) | **1.38 (1.26-1.51)** | **1.23 (1.13-1.35)** | **1.26 (1.15-1.38)** |
| ***Gender*** | | | | | |
| *Male* | | | | | |
| No confirmed diagnosis | 8377 | 413 (4.9) | Ref. | Ref. | Ref. |
| Ever diagnosed with COVID-19 | 1489 | 98 (6.6) | 1.31 (1.00-1.71) | 1.19 (0.91-1.57) | 1.20 (0.92-1.57) |
| *Female* | | | | | |
| No confirmed diagnosis | 34 607 | 3000 (8.7) | Ref. | Ref. | Ref. |
| Ever diagnosed with COVID-19 | 6136 | 784 (12.8) | **1.39 (1.26-1.53)** | **1.24 (1.12-1.36)** | **1.27 (1.15-1.39)** |
| ***Age groups (years)*** | | | | | |
| *18-39* | | | | | |
| No confirmed diagnosis | 10 915 | 1258 (11.5) | Ref. | Ref. | Ref. |
| Ever diagnosed with COVID-19 | 2441 | 367 (15.0) | **1.17 (1.01-1.35)** | **1.16 (1.01-1.34)** | **1.17 (1.01-1.35)** |
| *40-59* | | | | | |
| No confirmed diagnosis | 16 911 | 1603 (9.5) | Ref. | Ref. | Ref. |
| Ever diagnosed with COVID-19 | 3865 | 44 (11.6) | **1.22 (1.08-1.38)** | **1.22 (1.08-1.38)** | **1.27 (1.12-1.43)** |
| *60+* | | | | | |
| No confirmed diagnosis | 15 158 | 552 (3.6) | Ref. | Ref. | Ref. |
| Ever diagnosed with COVID-19 | 1319 | 67 (5.1) | 1.39 (1.00-1.94) | **1.40 (1.01-1.96)** | **1.42 (1.02-1.96)** |
| ***Education*** | | | | | |
| *Compulsory* | | | | | |
| No confirmed diagnosis | - | - | - | - | - |
| Ever diagnosed with COVID-19 | - | - | - | - | - |
| *Secondary or others* | | | | | |
| No confirmed diagnosis | - | - | - | - | - |
| Ever diagnosed with COVID-19 | - | - | - | - | - |
| *University or above* | | | | | |
| No confirmed diagnosis | - | - | - | - | - |
| Ever diagnosed with COVID-19 | - | - | - | - | - |
| ***Above threshold for depression (using the Patient Health Questionnaire with the recommended cutoff score of ≥10)*** | | | | | |
| *No* | | | | | |
| No confirmed diagnosis | 35 638 | 1362 (3.8) | Ref. | Ref. | Ref. |
| Ever diagnosed with COVID-19 | 6114 | 401 (6.6) | **1.69 (1.48-1.94)** | **1.51 (1.32-1.73)** | **1.53 (1.33-1.75)** |
| *Yes* |  |  |  |  |  |
| No confirmed diagnosis | 6873 | 1990 (29.0) | Ref. | Ref. | Ref. |
| Ever diagnosed with COVID-19 | 1434 | 469 (32.7) | 1.04 (0.93-1.17) | 1.04 (0.93-1.16) | 1.05 (0.94-1.17) |
| ***Previous psychiatric diagnosis*** | | | | | |
| *No* | | | | | |
| No confirmed diagnosis | 29 046 | 1092 (3.8) | Ref. | Ref. | Ref. |
| Ever diagnosed with COVID-19 | 5023 | 298 (5.9) | **1.49 (1.26-1.75)** | **1.30 (1.11-1.53)** | **1.32 (1.12-1.55)** |
| *Yes* | | | | | |
| No confirmed diagnosis | 13 022 | 2217 (17.0) | Ref. | Ref. | Ref. |
| Ever diagnosed with COVID-19 | 2443 | 562 (23.0) | **1.29 (1.15-1.44)** | **1.22 (1.10-1.37)** | **1.24 (1.11-1.38)** |
| ***Pre-existing comorbidity*** | | | | | |
| *No* | | | | | |
| No confirmed diagnosis | 27 959 | 1963 (7.0) | Ref. | Ref. | Ref. |
| Ever diagnosed with COVID-19 | 5322 | 580 (10.9) | **1.41 (1.25-1.58)** | **1.30 (1.15-1.46)** | **1.34 (1.19-1.50)** |
| *Yes* | | | | | |
| No confirmed diagnosis | 13 181 | 1204 (11.7) | Ref. | Ref. | Ref. |
| Ever diagnosed with COVID-19 | 1995 | 234 (11.7) | **1.37 (1.16-1.62)** | 1.10 (0.94-1.30) | 1.16 (0.99-1.36) |
| ***Year of COVID-19 diagnosis*** | | | | | |
| *2020* | | | | | |
| No confirmed diagnosis | - | - | Ref. | Ref. | Ref. |
| Ever diagnosed with COVID-19 | 2961 | 340 (11.5) | **1.50 (1.30-1.72)** | **1.37 (1.19-1.58)** | **1.42 (1.24-1.63)** |
| *2021* | | | | | |
| No confirmed diagnosis | - | - | Ref. | Ref. | Ref. |
| Ever diagnosed with COVID-19 | 3811 | 431 (11.3) | **1.37 (1.20-1.57)** | **1.21 (1.06-1.39)** | **1.26 (1.10-1.44)** |
| *2022* | | | | | |
| No confirmed diagnosis | - | - | Ref. | Ref. | Ref. |
| Ever diagnosed with COVID-19 | 853 | 111 (13.0) | **1.48 (1.23-1.78)** | **1.24 (1.04-1.47)** | 1.18 (0.98-1.42) |
| ***Number of COVID-19 diagnoses*** | | | | | |
| *1* | | | | | |
| No confirmed diagnosis | - | - | Ref. | Ref. | Ref. |
| Ever diagnosed with COVID-19 |  |  | **1.38 (1.26-1.52)** | **1.24 (1.13-1.36)** | **1.27 (1.16-1.39)** |
| *2 or more* | | | | | |
| No confirmed diagnosis | - | - | Ref. | Ref. | Ref. |
| Ever diagnosed with COVID-19 |  |  | 1.19 (0.66-2.15) | 0.93 (0.52-1.67) | 0.93 (0.53-1.65) |

^a^ Significant prevalence ratios are in bold

## **STROBE Statement—Checklist of items that should be included in reports of *cross-sectional studies***

|  | Item No | Recommendation | Page no. |
| --- | --- | --- | --- |
| **Title and abstract** | 1 | (*a*) Indicate the study’s design with a commonly used term in the title or the abstract | 1 |
|  |  | (*b*) Provide in the abstract an informative and balanced summary of what was done and what was found | 2 |
| Introduction | | |  |
| Background/rationale | 2 | Explain the scientific background and rationale for the investigation being reported | 3 |
| Objectives | 3 | State specific objectives, including any prespecified hypotheses | 3 |
| Methods | | |  |
| Study design | 4 | Present key elements of study design early in the paper | 3 |
| Setting | 5 | Describe the setting, locations, and relevant dates, including periods of recruitment, exposure, follow-up, and data collection | 3, Table S2 |
| Participants | 6 | (*a*) Give the eligibility criteria, and the sources and methods of selection of participants | 3 |
| Variables | 7 | Clearly define all outcomes, exposures, predictors, potential confounders, and effect modifiers. Give diagnostic criteria, if applicable | 3-4, Table S4 |
| Data sources/ measurement | 8* | For each variable of interest, give sources of data and details of methods of assessment (measurement). Describe comparability of assessment methods if there is more than one group | 3-5 |
| Bias | 9 | Describe any efforts to address potential sources of bias | 4-6 |
| Study size | 10 | Explain how the study size was arrived at | 4-6, Figure S1 |
| Quantitative variables | 11 | Explain how quantitative variables were handled in the analyses. If applicable, describe which groupings were chosen and why | 4-6, Table S4 |
| Statistical methods | 12 | (*a*) Describe all statistical methods, including those used to control for confounding | 4-6 |
|  |  | (*b*) Describe any methods used to examine subgroups and interactions | 4-6 |
|  |  | (*c*) Explain how missing data were addressed | 3-5, Figure S1 |
|  |  | (*d*) If applicable, describe analytical methods taking account of sampling strategy |  |
|  |  | (*e*) Describe any sensitivity analyses | 5-6 |
| Results | | |  |
| Participants | 13* | (a) Report numbers of individuals at each stage of study—eg numbers potentially eligible, examined for eligibility, confirmed eligible, included in the study, completing follow-up, and analysed | 6, Tables 1, S5, S6 |
|  |  | (b) Give reasons for non-participation at each stage | 4-5, Figure S1 |
|  |  | (c) Consider use of a flow diagram | Figure S1 |
| Descriptive data | 14* | (a) Give characteristics of study participants (eg demographic, clinical, social) and information on exposures and potential confounders | 6 |
|  |  | (b) Indicate number of participants with missing data for each variable of interest | Tables 1, S5, S6 |
| Outcome data | 15* | Report numbers of outcome events or summary measures | 6-8, Tables S7, S9 |
| Main results | 16 | (*a*) Give unadjusted estimates and, if applicable, confounder-adjusted estimates and their precision (eg, 95% confidence interval). Make clear which confounders were adjusted for and why they were included | Tables S7-S9 |
|  |  | (*b*) Report category boundaries when continuous variables were categorized | 6-8 |
|  |  | (*c*) If relevant, consider translating estimates of relative risk into absolute risk for a meaningful time period |  |
| Other analyses | 17 | Report other analyses done—eg analyses of subgroups and interactions, and sensitivity analyses | 7-10 |
| Discussion | | |  |
| Key results | 18 | Summarise key results with reference to study objectives | 10 |
| Limitations | 19 | Discuss limitations of the study, taking into account sources of potential bias or imprecision. Discuss both direction and magnitude of any potential bias | 10-12 |
| Interpretation | 20 | Give a cautious overall interpretation of results considering objectives, limitations, multiplicity of analyses, results from similar studies, and other relevant evidence | 10-12 |
| Generalisability | 21 | Discuss the generalisability (external validity) of the study results | 10-11 |
| Other information | | |  |
| Funding | 22 | Give the source of funding and the role of the funders for the present study and, if applicable, for the original study on which the present article is based | 13 |

*Give information separately for exposed and unexposed groups.

**Note:** An Explanation and Elaboration article discusses each checklist item and gives methodological background and published examples of transparent reporting. The STROBE checklist is best used in conjunction with this article (freely available on the Web sites of PLoS Medicine at http://www.plosmedicine.org/, Annals of Internal Medicine at http://www.annals.org/, and Epidemiology at http://www.epidem.com/). Information on the STROBE Initiative is available at www.strobe-statement.org.
